# A Diverse Population of *Cryptococcus gattii* Molecular Type VGIII in Southern Californian HIV/AIDS Patients

**DOI:** 10.1371/journal.ppat.1002205

**Published:** 2011-09-01

**Authors:** Edmond J. Byrnes, Wenjun Li, Ping Ren, Yonathan Lewit, Kerstin Voelz, James A. Fraser, Fred S. Dietrich, Robin C. May, Sudha Chatuverdi, Vishnu Chatuverdi, Joseph Heitman

**Affiliations:** 1 Department of Molecular Genetics and Microbiology, Duke University Medical Center, Durham, North Carolina, United States of America; 2 Mycology Laboratory, Wadsworth Center, Albany, New York, United States of America; 3 School of Biosciences, University of Birmingham, Birmingham, United Kingdom; 4 Centre for Infectious Disease Research, Department of Molecular and Microbial Sciences, University of Queensland, Brisbane, Australia; 5 Institute for Genome Sciences and Policy, Duke University Medical Center, Durham, North Carolina, United States of America; Carnegie Mellon University, United States of America

## Abstract

*Cryptococcus gattii* infections in southern California have been reported in patients with HIV/AIDS. In this study, we examined the molecular epidemiology, population structure, and virulence attributes of isolates collected from HIV/AIDS patients in Los Angeles County, California. We show that these isolates consist almost exclusively of VGIII molecular type, in contrast to the VGII molecular type isolates causing the North American Pacific Northwest outbreak. The global VGIII population structure can be divided into two molecular groups, VGIIIa and VGIIIb. Isolates from the Californian patients are virulent in murine and macrophage models of infection, with VGIIIa significantly more virulent than VGIIIb. Several VGIII isolates are highly fertile and produce abundant sexual spores that may serve as infectious propagules. The **a** and α VGIII *MAT* locus alleles are largely syntenic with limited rearrangements compared to the known VGI (**a**/α) and VGII (α) *MAT* loci, but each has unique characteristics including a distinct deletion flanking the 5′ VGIII *MAT* **a** alleles and the α allele is more heterogeneous than the **a** allele. Our studies indicate that *C. gattii* VGIII is endemic in southern California, with other isolates originating from the neighboring regions of Mexico, and in rarer cases from Oregon and Washington state. Given that >1,000,000 cases of cryptococcal infection and >620,000 attributable mortalities occur annually in the context of the global AIDS pandemic, our findings suggest a significant burden of *C. gattii* may be unrecognized, with potential prognostic and therapeutic implications. These results signify the need to classify pathogenic *Cryptococcus* cases and highlight possible host differences among the *C. gattii* molecular types influencing infection of immunocompetent (VGI/VGII) vs. immunocompromised (VGIII/VGIV) hosts.

## Author Summary

Infections that were once uncommon have become considerable public health threats due to the increased number of patients with weakened immunity, including a large HIV infected population. Among the emerging infections in the HIV/AIDS population are those caused by fungi. Fungi are distinct from viral or bacterial pathogens and often cause skin (athletes foot), mouth (thrush), or vaginal (yeast) infections. Life-threatening infections of the lungs and brain are caused by *Cryptococcus*, which infects >1,000,000 patients annually causing ∼1/3 of all AIDS-related deaths. Many infections are caused by *C. neoformans*, but the sibling species *C. gattii* infects both healthy individuals and HIV/AIDS patients and is causing an outbreak in the Pacific Northwest. Often, *Cryptococcus* is not identified at the species level, and *C. gattii* may therefore be more common than appreciated. We studied *C. gattii* isolates infecting HIV/AIDS patients in southern California and found they are unrelated to those causing the Pacific Northwest outbreak (molecular type VGIII rather than VGII). We show that within VGIII there are two subgroups, and one is more virulent than the other in animals. Our studies establish a foundation for research on *C. gattii* in HIV/AIDS patients. Given the global magnitude of infections, these findings have significant public health implications.

## Introduction

The pathogenic *Cryptococcus* species complex is comprised of two common fungal pathogens of humans and other animals: *C. neoformans* and *C. gattii* [1]. The more prevalent *C. neoformans* is ubiquitously distributed worldwide and a common cause of meningitis in immunocompromised hosts [1], [2], [3]. *C. gattii* is more geographically restricted to tropical and subtropical regions, associated with eucalypts, Douglas fir, and other trees, and has a greater predilection for infecting immunocompetent hosts [4], [5]. However, the geographic distribution of this species has been expanding, with an outbreak occurring in the Pacific Northwest region of North America [4], [5], [6], [7], [8], [9], [10], [11], [12]. *C. gattii* can be subdivided into two serotypes (B and C) [13] and four molecular types (VGI, VGII, VGIII, VGIV) that appear to represent genetically isolated cryptic species [14], [15], [16]. VGI and VGII cause the majority of cases in otherwise healthy hosts. VGIII and VGIV appear to more commonly infect immunocompromised patients, including those with HIV/AIDS, similar to *C. neoformans* [3], [5], [17], [18], [19].

Compared to *C. neoformans*, less is known about the epidemiology and ecology of *C. gattii*, especially molecular types VGIII and VGIV. VGIV is rare globally but has been reported to cause infections in sub-Saharan Africa AIDS patients [18], while VGIII has been isolated from a number of regions worldwide [14], [16]. The limited epidemiological data may be due to a lack of laboratory species distinction, particularly in *Cryptococcus* cases among HIV/AIDS patients, although sporadic *C. gattii* infections in HIV/AIDS patients have been reported from many global regions [18], [20], [21], [22], [23], [24], [25], [26], [27], [28], [29], [30].

There are limited accounts for HIV/AIDS or VGIII/VGIV associated *C. gattii* in North America. However, *C. gattii* has been reported in southern California among an HIV/AIDS patient cohort and one AIDS patient in Mexico, and *C. gattii* VGIII/VGIV have been reported in clinical cases from Mexico [21], [22], [31]. Additionally, there are three reported VGIII isolates from the Pacific Northwest outbreak [6], [7], [10]. These reports suggest that *C. gattii* may be endemic in western North America.

Here we compared isolates from HIV/AIDS patients in southern California [22] with a global collection. Based on MLST analyses, >93% (28/30) of the *C. gattii* isolate cohort are VGIII, and genotypic diversity is much greater than in the Pacific Northwest VGII outbreak. The genotypes cluster into two distinct groups (VGIIIa, VGIIIb). These findings suggest that VGIII may have been endemic in southern California for a longer period of time compared to the more clonal VGII Pacific Northwest population, or that VGIII is more actively recombining and/or mutating. The high levels of diversity, together with results suggesting recombination, support ongoing genetic exchange.

The VGIII lineage is highly fertile compared to other *C. gattii* molecular types [32]. Isolates from our cohort include both mating types (α and **a**), unlike the exclusively α mating type Pacific Northwest outbreak [7], [16]. This is noteworthy, as **a**-α mating can promote recombination and yield infectious spores [33], [34], [35], [36], [37], [38]. Sexual recombination is critical in evolution of eukaryotic microbial pathogens, including both parasites and fungi [39], [40], [41], [42], [43]. For these reasons, we extended the analysis of the VGIII group to include extensive mating assays to determine genotypes associated with high fertility. Additionally, sequenced and characterized the *C. gattii* VGIII *MAT* locus alleles. These findings expand on previous studies of the *Cryptococcus MAT* locus [44], [45], [46], [47], [48] yielding new insights into plasticity of this genomic region involving rearrangements, gene truncation, and loss, which may impact fertility.

While previous studies have shown *C. gattii* is highly virulent in mice, the focus has been on genotypes causing disease in otherwise healthy hosts [16], [49], [50], with no studies to date comprehensively examining virulence of VGIII. Here we examined murine virulence and macrophage intracellular proliferation for both the VGIIIa and VGIIIb. The isolates were also selected based on mating type criteria to examine what roles, if any, the *MAT* locus plays in VGIII virulence. This was of interest, as previous studies have shown virulence differences in *C. neoformans* congenic isolates that differ only at *MAT* [51], [52]. Our results demonstrate the VGIIIa lineage has increased levels of virulence compared to VGIIIb.

Our studies reveal a complex population structure within the highly fertile VGIII molecular type, suggesting recombination in both the United States and globally via opposite and/or same-sex mating. We posit that genetic exchange and the formation of infectious spores may be contributing to an underlying endemic level of *C. gattii* infections in southern California. Additionally, we show that these isolates are virulent in both murine and macrophage infection models [10]. Overall, the VGIII isolates from HIV/AIDS patients show decreased levels of virulence in comparison to the Pacific Northwest outbreak VGII isolates. This study highlights the need for isolate typing at a resolution sufficient to distinguish both species and molecular type. Accurate isolate identification will advance understanding of the epidemiology, ecology, and health burden of this emerging pathogen, with potential prognostic and therapeutic benefits for the clinical management of cryptococcal infections.

## Results

### Molecular type VGIII forms two distinct lineages that are the predominant cause of *C. gattii* infection in a southern Californian AIDS patient cohort

**Figure 1:**
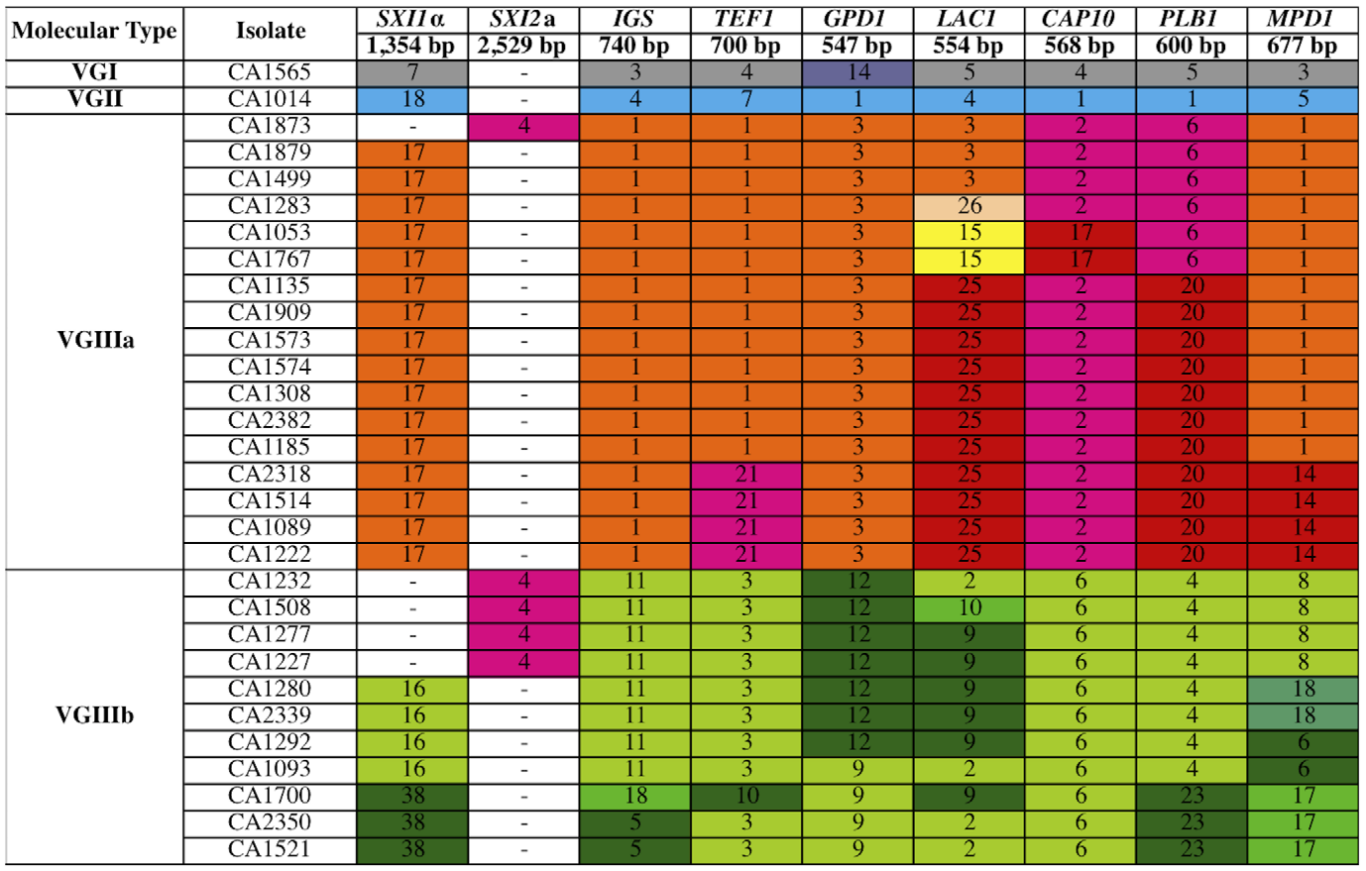
Molecular analysis of isolates from the Californian patient cohort reveals 93% are VGIII molecular type and genetically diverse. Multilocus sequence typing analysis of eight loci for the 30 isolates of *C. gattii* from the southern California AIDS patient cohort. Unique alleles are colored differently for each marker for visual discrimination, and each number represents a GenBank accession number (Table S4). Orange coloration represents VGIIIa, green VGIIIb, and fuchsia shared alleles. In total, a single isolate is VGI, a single isolate is VGII, and 28 isolates are VGIII molecular type.

**Figure 2:**
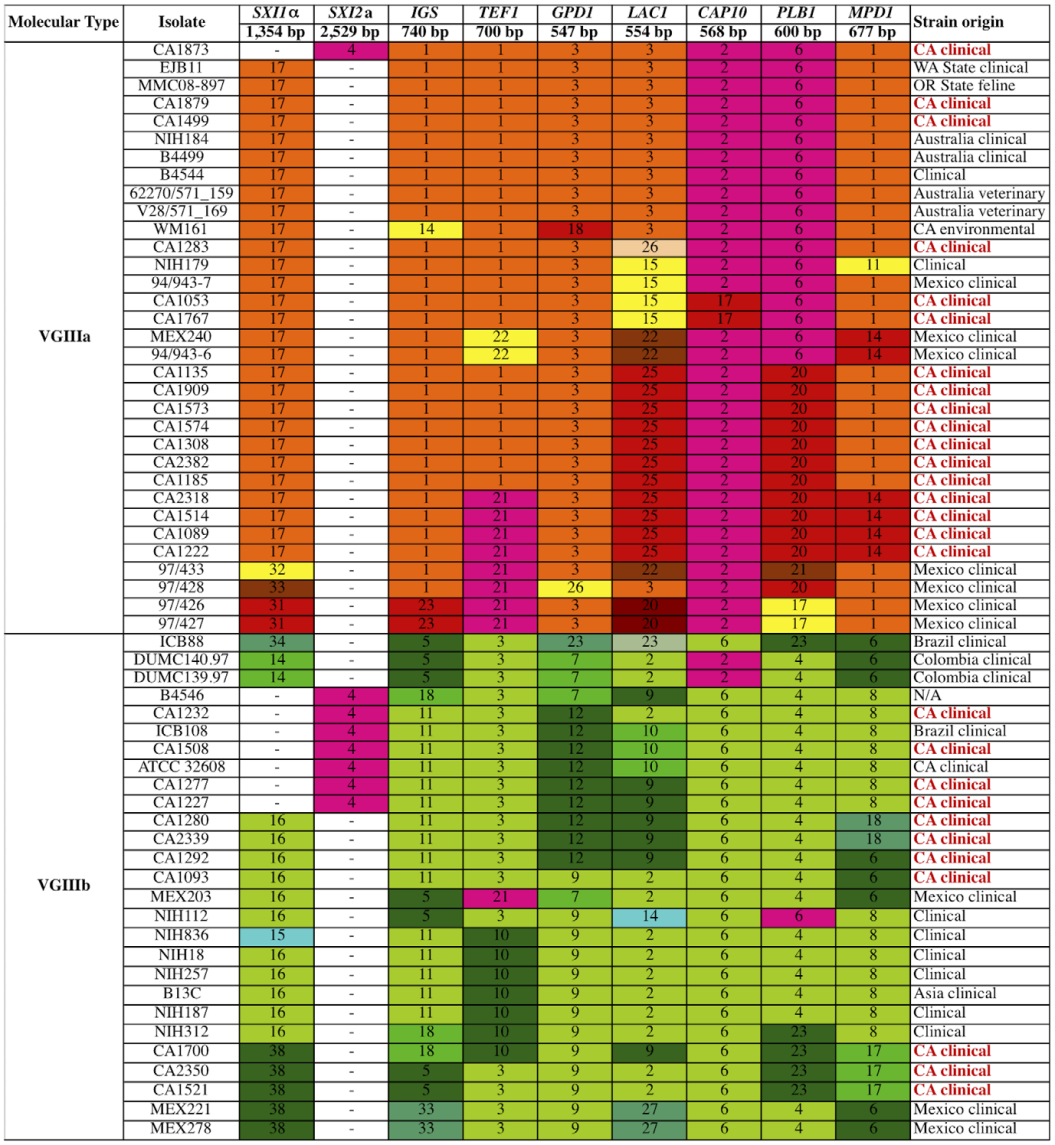
Global analysis of molecular markers illustrates high diversity and two distinct VGIII lineages: VGIIIa and VGIIIb. Multilocus sequence typing analysis was conducted at eight loci for the global collection of 60 VGIII molecular type isolates. Geographic origins of isolates are indicated, with red indicating isolates from the southern California patient cohort. Unique alleles were assigned distinct colors for each marker for visual discrimination, and each number represents a GenBank accession number (Table S4). Orange coloration represents VGIIIa, green VGIIIb, and fuchsia shared alleles.

To characterize the molecular type of *C. gattii* isolates collected from the southern California *C. gattii* cohort [22], we applied multilocus sequence typing (MLST) analysis at eight unlinked genomic loci. In total, the patient cohort consisted of 30 isolates. Of these, one was VGI (3.3%), another isolate was VGII (3.3%), and the remaining 28 isolates were molecular type VGIII (93.3%). Examination of the 28 VGIII isolates revealed a high level of diversity (13 unique genotypes based on seven MLST markers), isolates of the two mating types (α and **a**) (Figure 1), and no evidence for heterozygosity (consistent with FACS analysis showing they are haploid, data not shown). There are two genetically distinct groups within the VGIII molecular type. One was termed VGIIIa (predominantly orange shading in Figures 1, 2), and the other termed VGIIIb (predominantly green shading in Figures 1, 2). Additionally, two diploid isolates from the cohort (CA1388 and CA2355) were excluded due to high levels of heterozygosity in the majority of sequences, indicating they are likely hybrids. Hybrids between *C. neoformans* and *C. gattii* have been previously reported [53] (see discussion).

To further examine the isolates, their MLST profiles were compared to an additional 32 VGIII isolates collected from multiple locations and sources (Figure 2). Based on an examination of 60 VGIII isolates, the VGIIIa and VGIIIb clusters form two distinct groups. While many alleles were VGIIIa or VGIIIb specific, several were shared. Alleles shared between VGIIIa and VGIIIb are colored in fuchsia (Figures 1 and 2). In the VGIIIa cluster, all isolates originate from North America and Australia, while in the VGIIIb cluster, isolates originate from North America, South America, and Asia (Figure 2). Thus while there are possible geographic niches for each individual group, some geographic regions (Mexico and the US) harbor isolates from both groups. This finding suggests that in at least North America, the two groups might occupy similar environmental niches with potential for cross-hybridization.

**Figure 3:**
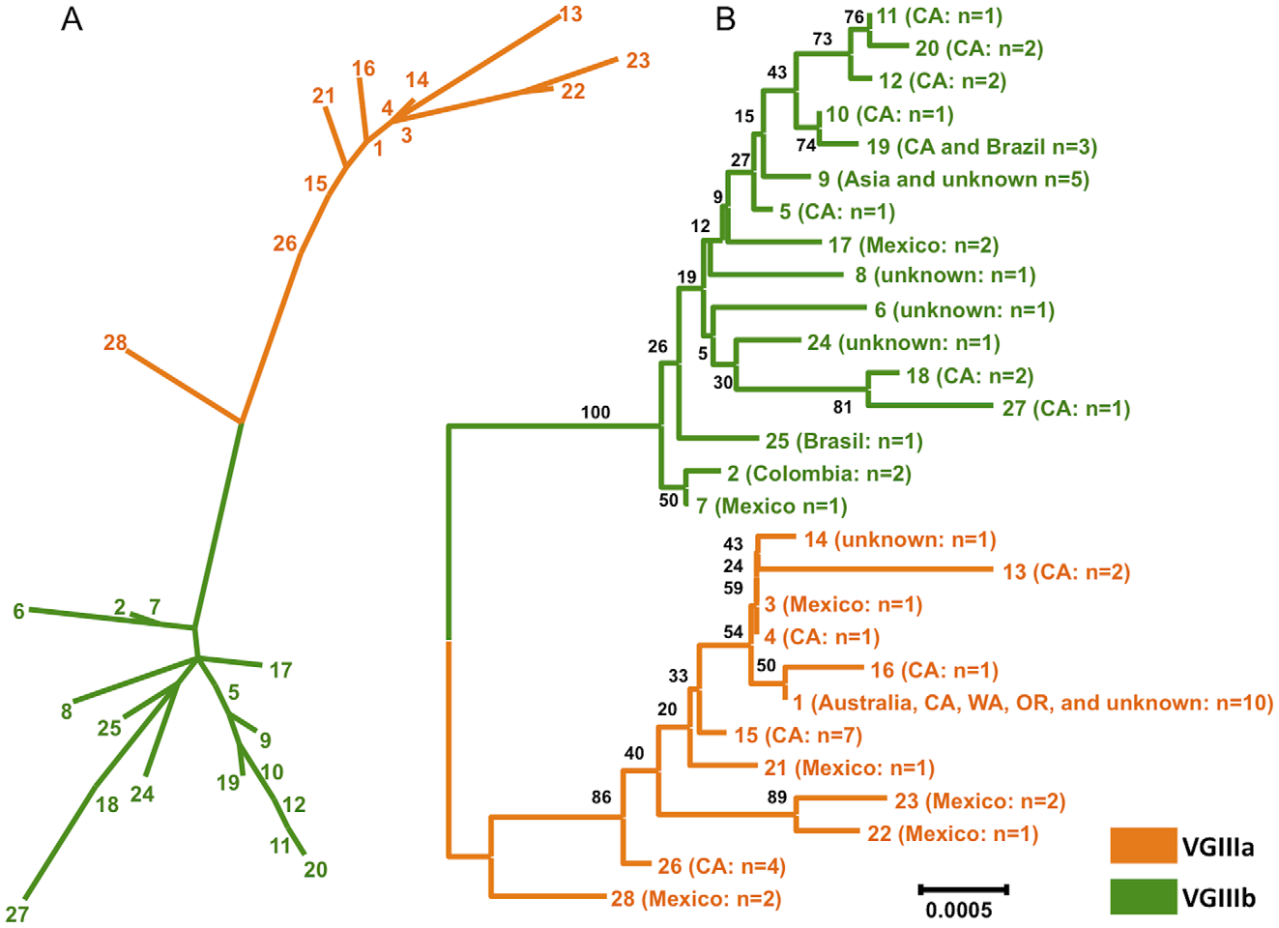
Clustering and phylogenetic analyses of global VGIII isolates reveals two well-supported lineages and one intermediate genotype. A) A clustering analysis representation (ML) of the concatenated sequence data from global isolates, with the exclusion of *MAT* locus linked markers (*SXI1*α/*SXI2* **a**). B) A phylogenetic representation (NJ) and supporting bootstrap values of the sequence data from global isolates, with the exclusion of *MAT* locus linked markers (*SXI1*α/*SXI2* **a**). In total, 28 unique sequence types were observed. Details of isolates within each sequence type are presented in Table S5.

We next examined the distribution of the sequence types by constructing maximum likelihood (ML) dendrograms (Figure 3A). This analysis also supports two distinct clusters. In total, 28 sequence types are represented in the seven-loci dendrogram 12 in VGIIIa and 16 in VGIIIb. One VGIIIa genotype, ST28, is an intermediate between the two subgroups, and consists of two clinical isolates from Mexico (97/426 and 97/427). This sequence type harbors two alleles shared between the two groups, two alleles common and exclusive to the VGIIIa group, and four alleles unique to this sequence type. The ancestry of this isolate remains unclear; however, both ST28 isolates originate from Mexico, a region that harbors isolates of both VGIIIa and VGIIIb, and thus these isolates may represent VGIIIa/VGIIIb hybrids.

The global population was examined by constructing Neighbor Joining phylograms (Figure 3B, Figure S1). This analysis also illustrates two defined lineages, with the ST28 genotype representing a VGIIIa/b intermediate. Bootstrap support for the separation of the VGIIIb lineage from the other genotypes is robust (100) (Figure 3B). Support for all VGIIIa genotypes other than ST28 is at a level of 86, indicating that ST28 may be a distinct lineage, or conversely be a divergent genotype within the VGIIIa lineage (Figure 3B). To address the ancestry of the VGIII subgroups, we examined the isolates in the context of the three other *C. gattii* molecular types (VGI, VGII, VGIV) (Figure S1). From this analysis, there is strong support (bootstrap value of 100) that the VGIIIa subgroup is ancestral to the VGIIIb subgroup, with ST28 as the closest genotype to the VGIIIb clade (Figure S1). While the two clusters (VGIIIa/VGIIIb) are distinct from one another, when compared with VGI, VGII, VGIV, all 60 VGIII isolates lie within the VGIII lineage, clearly delineated from the other molecular types with a bootstrap support of 100 (Figure S1).

### Speciation dynamics and evidence for recombination in the global VGIII population

**Figure 4:**
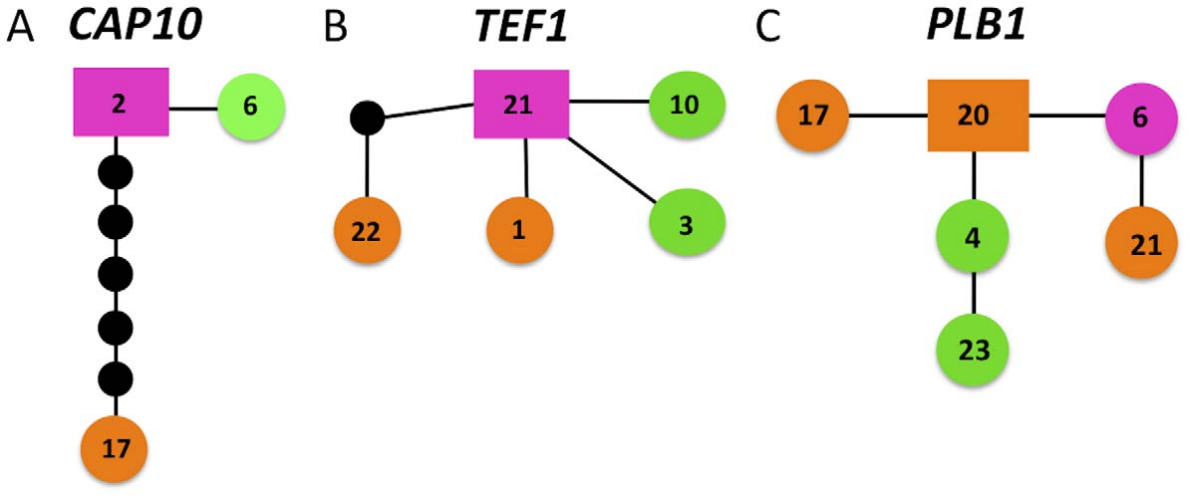
Haplotype mapping of markers harboring shared alleles between VGIIIa and VGIIIb shows evidence for both ancestral origins and introgression of shared alleles. Alleles for each respective locus are indicated numerically. Orange coloration represents VGIIIa, green VGIIIb, and fuchsia shared alleles. Circles represent alleles extant in the population, and the smaller black circles represent alleles that have not been recovered, or which may no longer be extant in the population. Each line connected to an object represents one postulated evolutionary event, with the squared allele representing the posited ancestral allele. A–C) Haplotype networks for *CAP10*, *TEF1*, and *PLB1*, respectively.

Based on an examination of the 60 total isolates, the VGIIIa and VGIIIb clusters may represent early speciation. To address aspects of the evolutionary history of the groups, haplotype network analysis was applied using TCS phylogenetic estimation for allele ancestry and evolution. The primary alleles focused on in this analysis were those shared between the subgroups. This examination addresses if shared alleles have a probable ancestral origin, or conversely if they were more recently introgressed. Of the shared alleles, *SXI2* **a** was not informative as there is only one allele. For the other three loci (*CAP10*, *TEF1*, *PLB1*), TCS analysis revealed that the *CAP10* locus and *TEF1* locus shared alleles appear ancestral, while the most parsimonious hypothesis is that the *PLB1* shared allele was introgressed between VGIIIa and VGIIIb because another allele (*PLB1-20*) is assigned as the ancestral root (Figure 4A–C).

In addition to markers encompassing shared alleles, haplotype networks were also constructed to examine the evolutionary history of the remaining five markers (Figure S2). For this analysis, four of the five markers (*LAC1*, *MPD1*, *GPD1*, and *IGS*) showed that the VGIIIa and VGIIIb subgroups are separated (Figure S2). This provides additional evidence for genetic isolation. The remaining marker, *SXI1*α, shows evidence for separation, but the history is less well resolved, and shared introgressed alleles may remain to be discovered in the population (Figure S2). This marker is highly variable and many of the proposed intermediates have not been found to date. This could be the result of: 1) under-sampling of isolates, 2) missing alleles that are either no longer extant in the population or never existed in cases where mutations occurred simultaneously, or 3) recombination within the allele. Overall, this analysis supports a genetic separation, with several hypothesized but likely rare introgression events having occurred.

**Figure 5:**
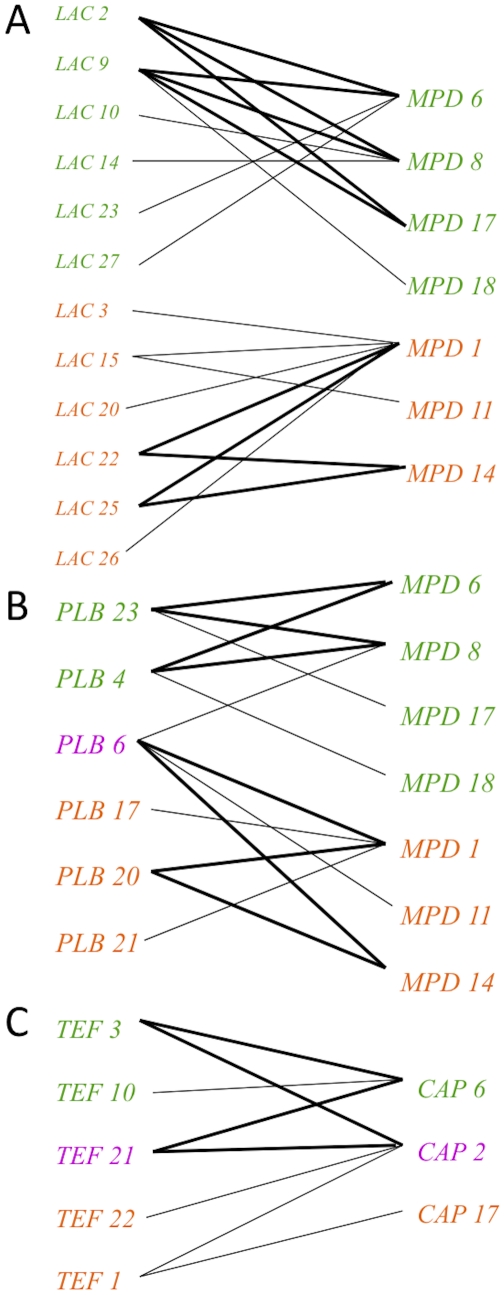
Evidence for genetic recombination within the VGIII population, particularly within alleles unique to the VGIIIa lineage. Informative paired allele graphs from VGIII global isolates. An hourglass shape indicates the presence of all four possible pairs of alleles and serves as evidence of recombination. A) An example of recombination evidence within VGIIIb (top) and VGIIIa (bottom). B) An example of recombination evidence within VGIIIb unique alleles (top) and an example involving a shared allele and three VGIIIa unique alleles (bottom). C) An example of recombination evidence involving both shared and VGIIIb unique alleles. All of the informative paired allele graphs constructed for this analysis can be found in Figure S3.

To examine the role that recombination may have contributed to population structure, we conducted a paired allele analysis among the global genotypes (Figure 5, Figure S3). The discovery of all four possible allele combinations between two unlinked loci (AB, ab, Ab, aB), in the absence of parallel mutations, serves as evidence for recombination [54], [55]. In total, 18 of 28 different molecular marker pairs showed evidence for recombination, with 25 examples in which all four allele combinations were observed. To further classify the analysis of the VGIIIa and VGIIIb subgroups, we examined how frequently allele combinations indicating recombination were associated with a specific lineage. In total, 1/25 locus pairs involved only the VGIIIa lineage, whereas 20/25 pairs involved only the VGIIIb lineage; the four remaining pairs involved shared alleles. Of the four pairs involving shared alleles, two involved shared and VGIIIa alleles and two involved shared and VGIIIb alleles. No pairs contained both VGIIIa and VGIIIb unique alleles (Figure 5, Figure S3). These studies demonstrate that while recombination may be present within both subgroups, the VGIIIb subgroup may be more actively undergoing recombination in its environmental niche.

To further characterize the role recombination may play in population structure, we employed MultiLocus software to examine the percentages of compatible loci and indices of association (I_A_). These analyses were completed for the global population, and also individually for the VGIIIa and VGIIIb subgroups. Additionally, the dataset was analyzed using all 60 isolates, and then was analyzed as a clone-corrected set with only unique genotypes represented (n = 28). Clone correction is commonly used to examine fungal populations that are known to often undergo asexual reproduction and clonal blooms, with less frequent sexual reproduction and meiosis [56]. The analysis of both the percentage of compatible loci and I_A_ produce p-values that if significant reject the null hypothesis of recombination. If the p-values are not significant, the null hypothesis cannot be rejected and recombination can be inferred. The analysis illustrates that although the null hypothesis is rejected in the analysis of all isolates, VGIIIa individual, VGIIIb individual, and the overall clone corrected dataset, the null hypothesis cannot be rejected in the analysis of the individual VGIIIa and VGIIIb subgroups when the dataset is clone corrected (Table 1). As we cannot reject the null hypothesis for recombination in the clone corrected subgroup analysis, it suggests recombination may be occurring within each subgroup. These results support the hypothesis of active recombination within the VGIII molecular type and that these events more likely occur between isolates within the same subgroup, rather than between subgroups, similar to studies supporting recombination in VGI and VGII populations in Australia [35], [36].

**Table 1:** Recombination analysis of the global VGIII population.

Population	n	Percent compatible loci	Percent compatible loci p value	Index of Association	Index of Association p value
All	60	0.29	<0.01	2.66	<0.01
VGIIIa	33	0.86	<0.01	0.67	<0.01
VGIIIb	27	0.38	<0.01	0.45	<0.01
All clone corrected	28	0.29	<0.01	1.78	<0.01
VGIIIa clone corrected	12	0.86	0.53	−0.01	0.47
VGIIIb clone corrected	16	0.38	0.72	0.13	0.28

**Figure 6:**
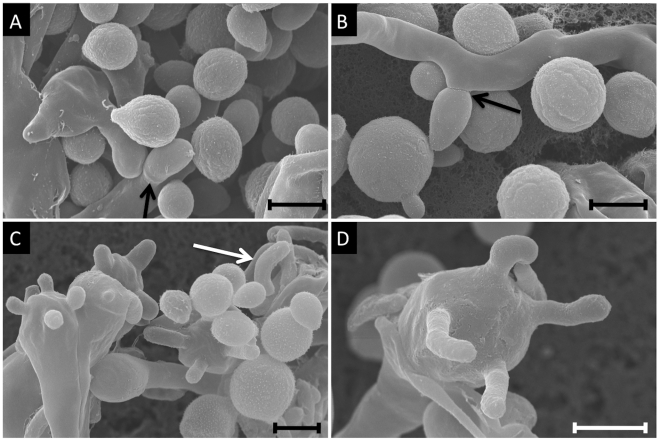
Scanning electron microscopy imaging of two VGIIIb isolates undergoing a-α mating to produce spores. Mating images are of NIH312α×B4546**a** from a V8 media (pH = 5) mating assay. A) Imaging of yeast cells, hyphae, and a clamp cell (arrow) (7,500×). B) Representation of yeast cells and hyphae, with an emerging bud seen to the left of the panel and a blastospore forming off of the hyphae (arrow, 7,000×). C) Yeast, hyphae, basidia, and basidiospores. One detached spore is seen in the top left of the panel, and is characteristically elongated (6,000×). D) A high magnification image of a single basidium with four emerging basidiospores (10,000×). All scale bars are 3 µm.

In addition to population-based studies, we conducted extensive mating assays among VGIII isolates, showing highly fertile isolates from both subgroups. In total, 182 mating pairs were examined by laboratory mating assays, including all eight mating type **a** isolates, and representatives from each of the α genotypes. When observed via light microscopy, 138 pairs showed no signs of fertility, while 44 pairs were able to undergo sexual reproduction, with representative high resolution SEM imaging of a VGIII×VGIII cross (NIH312×B4546) shown in Figure 6 (see also Tables 2 and S1). The SEM imaging, similar to previous studies [32], illustrated key hallmarks of mating including hyphae, fused clamp cells, basidia, and elongated basidiospores (Figure 6). When the strains that were fertile with the greatest number of mating partners were separated into the top four from each respective mating type, three of four from both mating types are from the VGIIIb subgroup (Table S1). This shows that while both groups are fertile, levels of fertility (based on the number of fertile partners) may be higher in the VGIIIb subgroup, consistent with the increased number of unique VGIIIb genotypes (n = 16 out of 27 isolates or 59%) compared to VGIIIa (n = 12 out of 33 isolates or 36%). Overall, a higher percentage of **a** isolates are fertile compared to α isolates (Table 2). When both mating types are combined VGIIIb shows an increased percentage of fertile isolates compared to VGIIIa (Table 2). These findings support the hypothesis that mating may also occur in the environment.

**Table 2:** Fertility of VGIIIa and VGIIIb lineages.

Lineage	Mating-type	# Fertile/total	% Fertile
VGIIIa	**a**	1/1	100
VGIIIa	α	10/32	31
VGIIIb	**a**	7/7	100
VGIIIb	α	10/20	50
All Isolates	**a**	8/8	100
All Isolates	α	20/52	38
VGIIIa	**a**+α	11/33	33
VGIIIb	**a**+α	17/27	63

### Characterization of the VGIII *MAT* locus alleles illustrates distinct features

**Figure 7:**
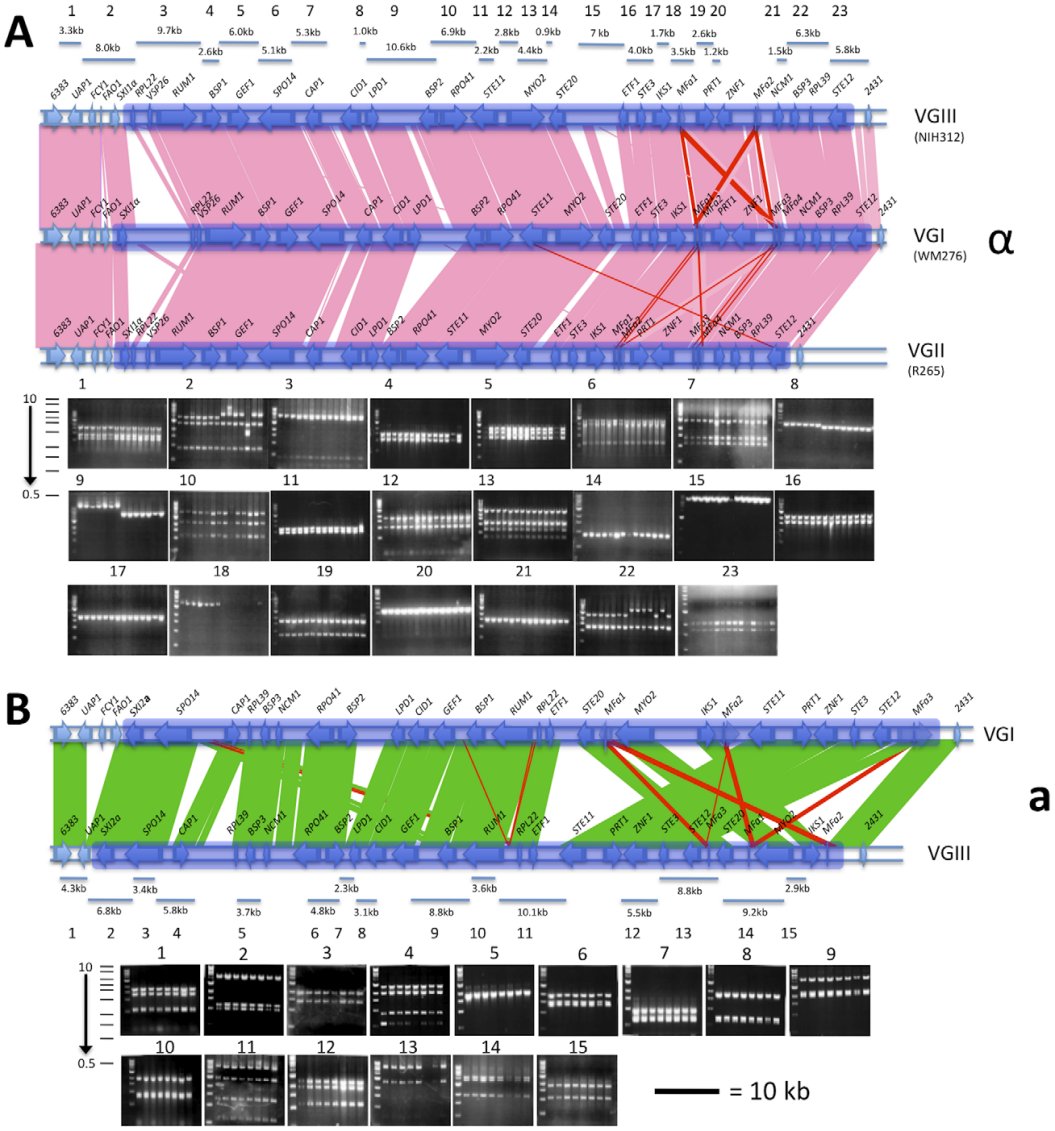
The VGIII *MAT* locus of α and a strains share relative synteny with other molecular types but also show rearrangements in *MAT*a. A) The *MAT* **a** locus and flanking regions of molecular type VGIII compared to the locus of molecular type VGI. Fingerprint markers are shown with the order of strains from left to right as follows: B4546, CA1227, CA1232, CA1277, CA1508, CA1873, ICB108, and ATCC32608. B) The *MAT*α locus and flanking regions of molecular type VGIII compared to the loci of molecular types VGI and VGII. Fingerprint markers are shown with the order of strains from left to right as follows: ICB88, DUMC140.97, CA1280, CA1093, NIH836 NIH312, CA1700, MMC08-897, WM161, MEX240, CA1514, 97/426, 97/433, and 97/428. The scale bar is equal to 10 kb and applies to both *MAT* locus alleles. Shading indicated the boundaries of the *MAT* locus alleles.

Sequencing of the α and **a** *MAT* locus alleles from two representative strains of the VGIII lineage shows that overall, the general structure, size, and characteristics are similar to previously sequenced *C. gattii* VGI and VGII *MAT* loci (Figure 7). Both *SXI1*α or *SXI2* **a** and the pheromone receptor and pheromone genes are present in the *MAT* locus, further supporting that *C. gattii* VGIII has a bipolar mating system.

Although there are marked similarities for the *MAT* locus alleles of VGI/VGII with VGIII, there are also distinct characteristics for each VGIII *MAT* allele. There are two rearrangements in the *MAT* **a** allele 3′ region compared to the VGI isolate E566. Moreover, a partial deletion of *UAP1* and loss of the *FCY1* and *FAO1* genes from the VGIII *MAT* **a** allele 5′ flanking region was identified via sequencing of the VGIII **a** isolate B4546 and confirmed to be present in all eight VGIII **a** isolates examined, although no phenotypic consequences were observed (Figures 7, S4, and S5). Southern blot analysis of *FCY1* and PCR analysis of all three full length genes with primers selected based on regions conserved between *C. neoformans* and *C. gattii* shows that they might not be present in the genome, or alternatively that they are rapidly evolving and too diverged to hybridize with probes and primers used (Figure S5). All isolates remain sensitive to the antifungal agent 5-Fluorocytosine (5-FC) indicating that the *FCY1* gene is functioning or that another gene acts in a similar manner. Additionally, an ∼1.1 kb truncation of the *FAO1* gene, a putative iron oxidoreductase, was found in all VGIII α strains analyzed. Using PCR, we could not detect an intact *FAO1* gene elsewhere in the genome of the VGIII α isolates. Four pheromone gene copies were previously found within the VGII and VGI *MAT*α loci, arranged in two pairs of divergently oriented, linked genes. However, although VGIII has been shown to be more fertile than VGI/VGII, based on the sequence assembly for strain NIH312, we found only two copies of the *MF*α pheromone gene, although one gap remains in this region of the locus. Additionally, we discovered a remnant of the **a** specific gene *SXI2* **a** in the VGIIIα mating type locus, and through sequence comparisons show that it is present in *C. gattii* molecular types VGI, VGII, and VGIII, but not present in *C. neoformans* var. *neoformans* (serotype D) or *C. neoformans* var. *grubii* serotype A (Figure S6).

Fingerprinting analysis of the *MAT* locus alleles revealed no size polymorphisms within *MAT* for VGIII **a** isolates, but did reveal diversity within the α allele (Figure 7, Table S2). Distinct alleles for fingerprint products 9 and 18 of the α locus, were identified that are correlated with the VGIIIa and VGIIIb molecular types (Figures 7, Table S2). Additionally, a 120 bp polymorphism within the *CID1* and *LPD1* intergenic sequence (fingerprint 8 of the α locus) was correlated with VGIIIa and VGIIIb molecular types with the exception of one VGIIIb strain harboring the *SXI*α allele 38, which contained the VGIIIa genotype at this region and could reflect recombination within *MAT* as a result of α-α mating (Figure 7, Table S2). Other VGIII isolates with *SXI1*α allele 38 were also confirmed to have this genotype (data not shown) and fingerprints 2 and 7 of the α locus showed multiple genotypes that were not correlated with the subgroups.

### Virulence and histopathology

**Figure 8:**
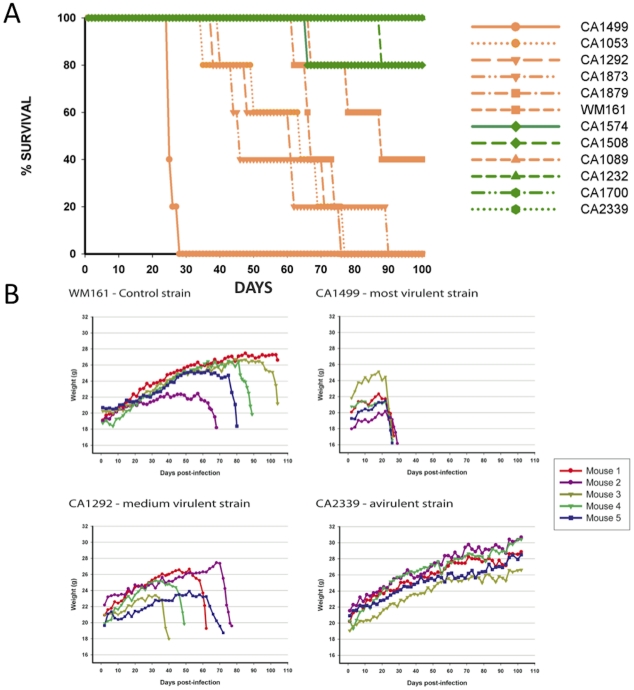
Isolates from the VGIIIa group exhibit increased virulence compared to VGIIIb in an in vivo murine model of infection. A) Groups of five animals were each infected with an infectious inoculum of 1.0×10^5^ cells, and the survival of animals plotted against days post-infection. The legend is arranged from most virulent (top) to least virulent (bottom, based on survival). VGIIIa isolates are indicated by orange coloration and VGIIIb isolates are indicated by green coloration. B) *C. gattii* infections cause notable weight loss associated with the virulence level of strains. BALB/c mice were infected with 1.0×10^5^ yeast cells via the intranasal instillation route. The weight of each animal was monitored every other day after infection while asymptomatic. When the animals began to exhibit symptoms of infection, weight was measured daily and plotted.

To address the virulence characteristics of the VGIII isolates, and correlate these with genotype and mating type, we conducted murine intranasal instillation challenges. In total, we chose 11 isolates from the patient cohort and one environmental VGIII control strain isolated in San Diego, California from an *E. camaldulensis* tree in 1992 [57]. Of the eleven clinical isolates, six were from the VGIIIa subgroup and five from the VGIIIb subgroup, with eight α and three **a** isolates (Figure 8A). Of the isolates examined, CA1499 was more virulent than all other isolates tested (Figure 8A). Additionally, when we compared overall virulence between the two subgroups, the VGIIIa subgroup showed a significantly higher mortality rate when compared to the VGIIIb subgroup (p<0.025). When isolate CA1089 was excluded from this analysis, the support increased (p<0.005). These results suggest that the molecular differentiation between the two lineages within VGIII is associated with a dichotomy in murine mortality post cryptococcal infection. We also show that the environmental isolate WM161 (VGIIIa) was less virulent than the five clinical VGIIIa isolates, but more virulent than all of the VGIIIb isolates. Isolates with increased levels of mortality were also found to be associated with rapid declines in total body weight during the course of infection (Figure 8B).

**Figure 9:**
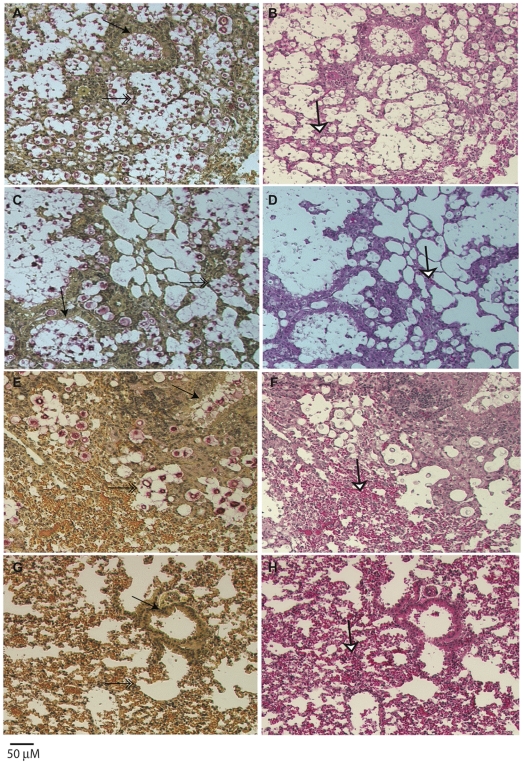
Histopathology of lungs of mice infected with *C. gattii*. Male, BALB/c mice were infected with 1×10^5^ yeast cells via intranasal instillation. The infected lungs were harvested at two weeks post-infection and processed as described above. Note that airway (arrow, A) and all the alveoli (double arrow, A) were packed with rapidly dividing yeasts with minimum tissue response (white arrow, B) in mice infected with highly virulent strain CA1499, while moderately virulent strain CA1292 showed a similar pattern except that several empty alveoli surrounding infected alveoli were observed (C, D). In contrast, the low virulene strain WM161 revealed less multiplication of *C. gattii* with a majority of alveoli containing one or two yeasts with an influx of inflammatory response (E, F). No yeast was seen in the lungs of mice infected with avirulent strain CA2339 and a vigorous inflammatory response was visible throughout the lung section (G, H). (Mucicarmine and H & E staining, ×100 magnification, scale bar 50 uM).

Histological examinations of lung tissues from mice infected with the highly virulent *C. gattii* strain CA1499 revealed widespread dissemination of cryptococci throughout the alveoli and airways with little to no inflammatory response. The expansion of alveoli with confluent clusters of budding yeasts and breakdown of alveolar walls were seen (Figure 9A–9B). The lungs of mice infected with strain CA1292 (moderate virulence) also revealed minimum inflammation with alveoli engorged with rapidly dividing yeasts. Also evident were a few alveoli with one to two *C. gattii* yeasts. Despite rapid multiplication, this strain appeared to be less disseminated as some of the alveoli and airways were devoid of any yeast cells (Figure 9C–9D). In contrast, lungs of mice infected with the low virulence strain WM161 revealed influx of inflammatory response surrounding infected alveoli and airways. The majority of the infected alveoli contained only one to two yeasts with a few alveoli containing three or four yeasts (Figure 9E–9F). No yeast cells were seen in any of the lung sections of mice infected with avirulent strain CA2339; however, vigorous tissue response in the form of neutrophil influx was observed throughout the lung section (Figure 9G–9H). The brain sections of all infected mice did not reveal any apparent lesions except for the highly virulent strain (CA1499), which showed occasional single or budding yeasts on the meninges (data not shown).

**Figure 10:**
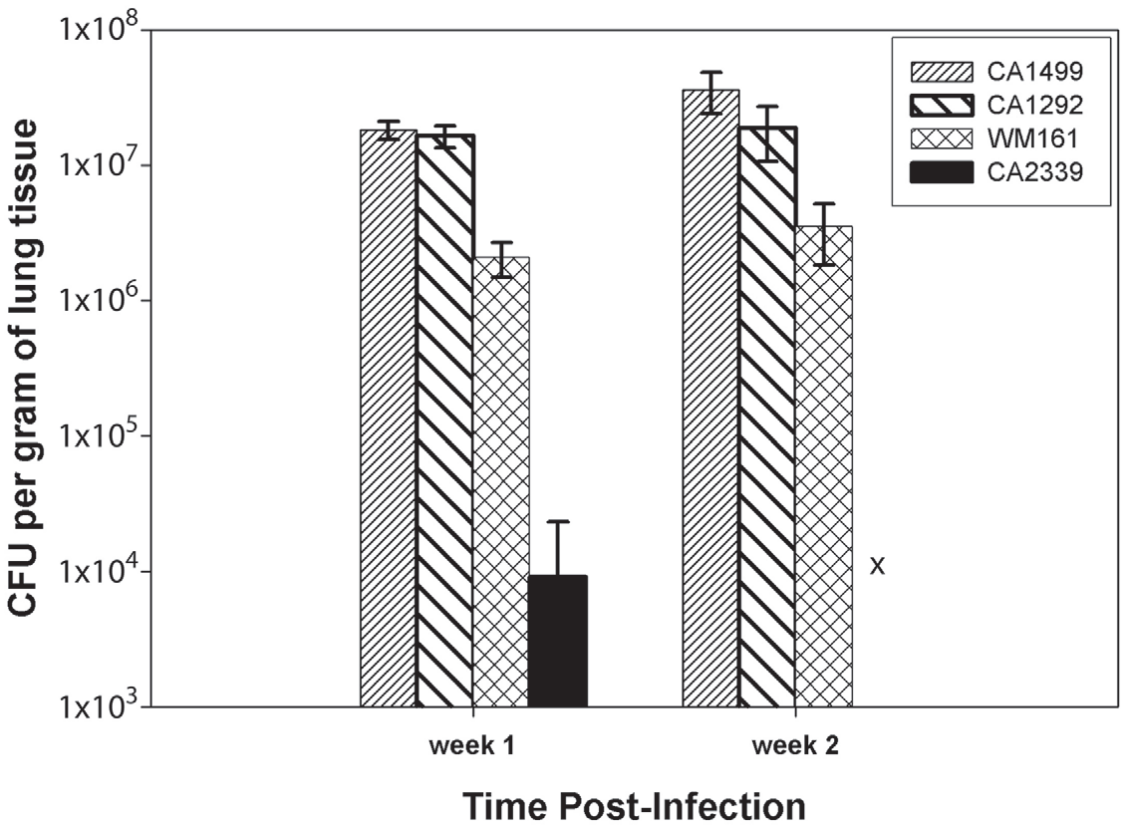
*C. gattii* organ load determination. Male, BALB/c mice were infected intranasally with 1×10^5^ *C. gattii* yeast cells intranasally. After one and two weeks post-infection, lungs and brains from three infected mice were removed asceptically and fungal load was determined by CFU enumeration as described in materials and methods. The bar diagram represents mean ± SD of CFU per gram of lung tissues obtained from three mice each for different *C. gattii* strains.

Although there were some differences in the dissemination pattern of CA1499 (high virulence) and CA1292 (moderate virulence) by histopathology, the quantification of yeasts from infected lungs at weekly intervals did not reveal any significant difference (Figure 10). This could be due to heavy colonization by CA1292 in the infected sites (Figure 9C–9D). When the lung organ loads of these two strains were compared with that of lung organ load of mice infected with low virulent strain WM161, the results were statistically significant (P<0.05). In comparison, the avirulent strain was cleared rapidly by one week post-infection and was not found in the lungs at two weeks post-infection (Figure 10). Overall, these results indicated that *C. gattii* rapid multiplication, dissemination, and invasion of the host defense of the lungs dictate the outcome of pulmonary cryptococcosis. Only small numbers of yeasts were recovered from brain cultures of mice infected with virulent strains of *C. gattii* (data not shown). These results indicated that pulmonary cryptococcosis and not CNS dissemination was the primary disease manifestation in this model and the likely cause of mice mortality observed (Figure 8).

**Figure 11:**
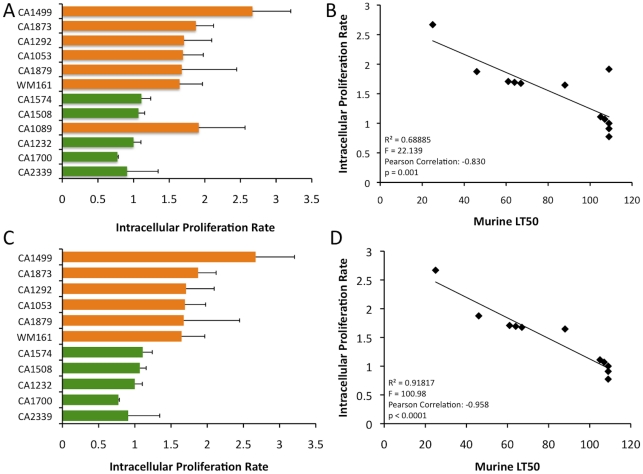
In vitro analysis of intracellular proliferation rates (IPR) show increased proliferation levels in VGIIIa and IPR values correlate with in vivo virulence. A) IPR values for the 12 isolates examined in mice; orange indicates VGIIIa and green indicates VGIIIb. B) Regression analysis illustrating a linear correlation of IPR and the time to 50% lethality in mice. C) IPR values for the isolates examined in mice, CA1089 excluded; orange indicates VGIIIa and green indicated VGIIIb. D) Regression analysis illustrating a linear correlation of IPR and the time to 50% lethality in mice with isolate CA1089 excluded.

Intracellular proliferation rates (IPRs) were determined for each strain examined in the murine model of infection. This assay is correlated positively with murine virulence, based on previous studies Pacific Northwest outbreak isolates [10], [49]. To further examine the virulence characteristics for the VGIII isolates, intracellular proliferation of cells within macrophages was directly tested for the seven VGIIIa and five VGIIIb isolates that were examined in the mouse model (Figure 11 A–B). Similar to the murine experiments, the VGIIIa subgroup showed significantly higher IPR levels than the VGIIIb subgroup (p<0.005, Figure 11 A, C). Additionally, as in the previous studies, IPR values and survival time in vivo showed strongly positive correlations upon regression analysis (Figure 11 B, D). A single VGIIIa isolate, CA1089, was an outlier with high IPR but low virulence. Therefore, the regression analysis was conducted both with (Figure 11 A, B) and without this isolate (Figure 11 C, D). While the significance levels were better supported when CA1089 was excluded, they were statistically significant in both cases (Figure 11 B, D). The IPR analysis combined with the in vivo model shows that the isolates from the Californian patient cohort show virulence differences in which the VGIIIa subgroup was more virulent overall than the VGIIIb subgroup.

## Discussion

Globally the burden of cryptococcal disease is significant with approximately one million annual cases [19]. Although >99% of AIDS related infections and >95% of overall cases are attributed to *C. neoformans* serotype A [1], *C. gattii* also has been shown to cause disease among AIDS patients in both sub-Saharan Africa and the US [18], [22]. In the study of Litvintseva et al., an African AIDS cohort was found to be infected with *C. gattii* serotype C VGIV molecular type [18]. Here *C. gattii* isolates were examined from a previously reported HIV/AIDS patient cohort in southern California [22], and found to be almost exclusively (93%) VGIII molecular type. The finding of *C. gattii* VGIII and VGIV isolates associated with HIV/AIDS patients is in stark contrast to numerous *C. gattii* infections among immunocompetent individuals caused by VGI/VGII isolates, and the ongoing VGII outbreak in the North American Pacific Northwest [5], [7], [9], [10], [16], [58], [59], [60], [61], [62].

The Californian HIV/AIDS patient cohort *C. gattii* strains were analyzed in the context of a global VGIII isolate collection to further characterize this molecular type. Analysis of 60 isolates at eight loci resolved two distinct subgroups designated VGIIIa and VGIIIb. In contrast to the Pacific Northwest VGII outbreak that is largely clonal with one genotype causing the majority of illness [9], [10], [16], [63], based on analysis of seven non sex-linked loci we found a total of 13 unique genotypes in the 28 VGIII isolates from the Californian patients. This increased level of diversity suggests this molecular type was introduced into the region longer ago than the VGII lineage to the Pacific Northwest, or alternatively that the VGIII population in California is more rapidly diverging. Our studies, in addition to a recent study documenting VGIII isolates in Mexico, lead to the hypothesis that the VGIII molecular type may be endemic to a large area of western North America [16], [31]. These findings are significant, as there may be unrecognized cases of *C. gattii* among HIV/AIDS patients.

Phylogenetic analysis of the molecular types revealed that VGIIIa is basal to VGIIIb (Figure S1). Given that VGIIIa is more virulent in mice and murine macrophages, it may be that VGIIIb has become more specialized to infect hosts with increased susceptibility to infections. It currently remains unclear as to whether these two groups have naturally evolved to display differences in virulence or if there is some form of selection. Regardless, increased surveillance of VGIII cases, aided by clinical studies to determine the types of infection each group causes, should shed further light as to whether these two groups also have distinct clinical features. Clustering and phylogenetic analyses of the VGIII global collection revealed 28 unique genotypes among 60 isolates. Subsequent phylogenetic analysis revealed that the two observed groups were indeed well supported lineages. Haplotype mapping was then conducted, supporting the discrimination into VGIIIa and VGIIIb. Additionally, the mapping of markers harboring shared alleles between the subgroups revealed that two of the three shared alleles are ancestral in origin, while one may result from introgression between VGIIIa and VGIIIb. This type of introgression event is not unprecedented between distinct lineages, cryptic species, or species within the fungal kingdom, with examples from model fungi and both plant and animal pathogens [64], [65], [66], [67].

The ability for microbial pathogens to undergo sexual reproduction involving meiosis is unique to the eukaryotic lineage, including the parasites and fungi. Sex may play a significant role in several aspects of pathogenesis, including the generation of genetic diversity, the formation of invasive hyphae or spores, and direct links between the mating type locus and mating pathways with virulence [34], [35], [37], [39], [52], [68], [69], [70], [71], [72], [73], [74], [75], [76]. For these reasons, and to understand the population dynamics of VGIII, we conducted two types of recombination analyses: informative paired allele graphs and statistical analyses of percentages of compatible loci and I_A_. Our analyses support the hypothesis of recombination within the distinctive VGIII subgroups. This is consistent with the observed molecular differences between the two groups. Our population results, taken together with an increased number of highly fertile VGIIIb isolates in comparison to VGIIIa, suggest that the VGIIIb group may be more actively recombining. Furthermore, the two excluded hybrid isolates appear to be *C. neoformans* var. *neoformans/C. gattii* molecular type VGIIIb hybrids (based on the *GPD1* MLST allele specifically amplifying *C. gattii* sequence and the *IGS* allele specifically amplifying *C. neoformans* sequence, while the other MLST loci were highly heterozygous). The enhanced fertility ofn the VGIIIb subgroup may contribute to hybridization with *C. neoformans*.

The ability to undergo sexual reproduction also has implications for the formation of infectious spores. Recent studies show that spores can initiate disseminated cryptococcal disease in the murine inhalation model of infection [34], [37]. Laboratory studies have also shown that *Cryptococcus* can complete a full sexual cycle in association with plants, leading to the production of infectious spores [77]. It has been previously shown that the VGIII lineage shows high levels of fertility [32] and our examination of compatible isolate pairs supports that mating and spore formation may play a significant role in the formation of small aerosolized particles that could be readily inhaled. Furthermore, in California, both mating types are present and several of these pairs are fertile under laboratory conditions.

The *MAT* alleles of *C. gattii* molecular types VGI and VGII are highly conserved, based on both DNA sequence similarity and gene synteny [48]. The sequenced *MAT*α locus alleles of VGI WM276, VGII R265, and VGIII NIH312 share full synteny (Figure 7). A comparison of the three loci reveals only expansions, contractions, and translocation of intergenic sequences, which account for the majority of the structural variation. Additionally, there is also a truncation of the *FAO1* gene in all VGIII α isolates examined. The sequenced *MAT* **a** locus alleles of VGI E566 and VGIII B4546 show a higher degree of rearrangement in comparison to *MAT*α (Figure 7). Additionally, a partial deletion of the *UAP1* gene, along with a complete deletion of the *FCY1* and *FAO1* genes from the 5′end of the VGIII *MAT* **a** locus is fixed in the VGIII **a** population. *UAP1* is predicted to encode a candidate uric acid transporter whose closest homolog is the *A. nidulans uapA* gene [78], [79], [80] and Fcy1 is a cytosine deaminase that has been shown to confer resistance to 5-FC when deleted in *C. albicans*, *S. cerevisiae*, and *C. neoformans* [81], [82]. Although both PCR and southern blot analyses could not detect a copy of the *FCY1* gene in the genome of the VGIII *MAT* **a** isolates, phenotypic tests showed these isolates remain sensitive to 5-FC and they may therefore harbor a more diverged *FCY1* gene elsewhere in the genome. Phenotypic assays on media with uric acid as a sole nitrogen source to examine the possible loss of *UAP1* also showed no distinct phenotype from control isolates, i.e., all isolates were able to efficiently grow (data not shown). Based on findings in *C. neoformans*, recombination hotspots may flank the *MAT* locus alleles and thus have contributed to the flanking region deletions [83]. Overall, analysis of the *C. gattii MAT* locus alleles showed a more dynamic *MAT* **a** locus compared to the α locus. Given the significant rearrangement of the *MAT* **a** locus, and the deletion or exclusion of genes from the locus and flanking region, it appears that the VGIII *MAT* **a** population has experienced increased genetic variation. Deletion or exclusion of genes from the *MAT* locus and flanking regions may be an indication of expansion or contraction of the VGIII *MAT* **a** locus. The finding of a *SXI2* **a** remnant conserved in *C. gattii* may signal a previous tetrapolar mating type state, consistent with the prevailing models and studies in related *Cryptococcus* species (Figure S6 B) [46], [47], [48], [84], [85], or result from a *C. gattii* lineage specific gene conversion of *SXI2* **a** into the α allele (Figure S6 C). Alternatively, this remnant may be functional and influence mating.

Within VGIII, fingerprinting analysis revealed a monomorphic *MAT* **a** allele and a mostly biallelic *MAT*α locus correlating with the VGIIIa and VGIIIb lineages. Two fingerprints revealed size polymorphisms correlated with VGIIIa and VGIIIb isolates, serving as additional evidence for ongoing genetic separation between these two groups. VGIIIb strains harboring *SXI*α allele 38 contained the VGIIIa genotype for the intergenic sequence between *CID1* and *LPD1*, suggesting that these strains harbor either the ancestral VGIII *MAT*α allele or contain a hybrid *MAT*α locus that may be the result of a gene introgression event between the two groups or a recombination event between same-sex isolates of opposite VGIII lineages (i.e., VGIIIa/VGIIIb). However, an additional polymorphic marker within the *MAT*α locus is needed to address the latter hypothesis.

Following the definition of two well-supported lineages including isolates from both mating types, we examined virulence characteristics associated with these properties. We found significant differences between phylogenetic subgroups. In both whole animal murine in vivo intranasal instillation and proliferation assays in murine derived macrophages, the VGIIIa subgroup is more virulent than the VGIIIb subgroup. These findings are significant and serve as a foundation for future studies to determine the molecular basis for these observed phenotypes. Additionally, from a public health and epidemiological standpoint, it may be useful to determine if isolates are VGIIIa or VGIIIb. Clinical studies would have to be conducted in coordination to ascertain if the molecular subgroup is associated with altered clinical manifestations or outcomes. Based on the histological examinations, our findings indicate that host tissue responses in combination with yeast cell multiplication and capsule induction are associated with the outcome of pulmonary cryptococcosis, similar to previous studies documenting that upon serial in vivo passage of *C. neoformans*, strains increase in virulence with an associated decrease in capsule size [86].

Our study provides a comprehensive molecular and phenotypic overview of the *C. gattii* VGIII molecular type, which has historically been less studied than both the VGI and VGII molecular types. Our findings support two distinct lineages that might each be recombining, with the VGIIIa lineage showing higher levels of virulence in the models examined. Of significance, many of the isolates examined were from a cohort of HIV/AIDS patients in southern California. The high level of VGIII observed is in stark contrast to the Pacific Northwest VGII outbreak of VGII, in which the vast majority of cases reported are not associated with HIV/AIDS infected patients [7], [9], [10], [16], [63]. This suggests that *C. gattii* may occur in two general patient settings: VGI/VGII in otherwise healthy hosts (>50%) or those treated with steroids, vs. VGIII/VGIV predominantly in HIV/AIDS patients. Moreover, *C. gattii* infections may cause a substantial unrecognized health burden. To address these aspects, both retrospective and prospective studies should be conducted to: 1) survey global isolate collections from HIV/AIDS patients and 2) assign species and molecular types to newly collected isolates from HIV/AIDS patients with cryptococcal infections.

## Materials and Methods

### Phenotypic identification assays

All isolates from southern California and other global isolates were screened to confirm that they were *C. gattii.* Melanin production was assayed by growth and dark pigmentation on Staib's niger seed medium; urease activity was detected by growth and alkaline pH change on Christensen's agar. These tests established that isolates were *Cryptococcus* (*C. neoformans* or *C. gattii*). Additionally, isolates were assayed for resistance to canavanine and utilization of glycine on L-canavanine, glycine, 2-bromothymol blue (CGB) agar. Growth on CGB agar indicates that isolates are canavanine resistant, and able to use glycine as a sole carbon source, triggering a bromothymol blue color reaction indicative of *C. gattii*. All CGB positive isolates were then grown under rich culture conditions prior to genomic DNA extraction and storage at −80°C in 25% glycerol. For genomic DNA isolation, the MasterPure Yeast DNA purification kit from Epicentre Biotechnologies was used.

### Multilocus sequence typing

Each VGIII isolate examined in the analysis was subjected to multilocus sequence typing (MLST) [87] at a total of eight loci (Table S3). This marker set was selected to include loci that have been validated in other analyses of *C. gattii* [7], [16], [88], [89], [90]. For each isolate, genomic regions were PCR amplified, purified (ExoSAP-IT, Qiagen), and sequenced. Sequences from both forward and reverse strands were assembled with complete double strand coverage and manually edited using Sequencher version 4.8 (Gene Codes Corporation). Based on BLAST analysis of the GenBank database (NCBI), each allele was assigned a corresponding number, or given a new number if the sequence was not already in the database. GenBank accession numbers with corresponding allele numbers are listed in the supplementary information (Table S4).

### Clustering and phylogenetic analyses

For each isolate, a sequence type was defined as a sequence exhibiting a unique sequence profile, based on concatenation of the MLST markers. Each sequence type was confirmed to be unique by BLAST analysis of the NCBI GenBank database [91]. A multiple alignment of the sequences was carried out using Clustal W software [92]. Clustering analysis of the sequences was conducted using PhyML, which applies the maximum likelihood model for analysis [93]. The phylogenetic analysis was conducted using the Neighbor Joining algorithm. Haplotype network modeling was conducted using TCS software (version 1.21) [94]. The statistical recombination analysis was completed using MultiLocus software (version 1.2.2).

### Mating assays

Mating assays were conducted on V8 media (pH = 5). Isolates were incubated at room temperature in the dark for 2–4 weeks in dry conditions. Fertility was assessed by light microscopic examination for hyphae, fused clamp cells, basidia, and basidiospore formation at the periphery and surface of the co-incubated mating patch. All mating assays were conducted in duplicate. If there were no signs of fertility after the four-week period, isolate pairs were scored as having no fertility when paired together.

### Scanning Electron Microscopy (SEM)

SEM analysis was completed using protocols similar to those previously published [34]. Mating cultures (NIH312×B4546) were excised from V8 medium agar plates and fixed at 4°C in 3% glutaraldehyde that was buffered in 0.1 M Na cacodylate (pH = 6.8). Samples were then washed in triplicate using cold 0.1 M Na cacodylate buffer. This was followed by a graded dehydration series of 1 hr changes in cold 30% and 50% ethanol and held overnight. Dehydration was completed with 1 hr changes of cold 95% and 100% ethanol at 4°C and warming to room temperature in 100% EtOH. Two additional 1 hr changes of room temperature 100% EtOH completed the dehydration series. The samples were then critical point dried in liquid CO_2_ (Samdri-795; Tousimis Research Corp., Rockville, MD) for 15 min at the critical point. The agar pieces were mounted and sealed with silver paint to ensure good conductivity. The samples were sputter coated with 50 Å of Au/Pd (Hummer 6.2; Anatech U.S.A., Hayward, CA). Samples were held under vacuum conditions until viewed with a Jeol JSM 5900LV scanning electron microscope at 15 kV.

### Sequencing and analysis of the *MAT* locus alleles

The strains used for the construction of the Bacterial Artificial Chromosome (BAC) and fosmid libraries and analysis of the *MAT* locus alleles were NIH312 (*MAT*α) and B4546 (*MAT* **a**). Isolates were grown on YPD media at 30°C, and library construction was performed according to previous studies [48], [84]. Sequencing reactions were performed using BigDye 3.1 (Applied Biosystems, Foster City, California, United States) and analyzed on an ABI3100 sequencer. Sequence reads were assembled using the PHRED/PHRAP/CONSED package [95], [96] and Sequencher 4.8 (Gene Codes Corporation). Additional analysis of the data was performed using BLASTn [91]. Based on the initial assembly of sequences, oligonucleotides were selected to close gaps in the sequence coverage by primer walking. Genes on the *MAT* locus of *C. gattii* were annotated based on homology to the existing annotation in *C. gattii* VGII (strain R265) and VGI (strains WM276 and E566), and *C. neoformans* (H99 and JEC21). Certain gene annotations were modified using the FGENESH program (http://linux1.softberry.com/berry.phtml). Comparison of the *MAT* locus alleles among VGI, VGII, and VGIII strains was performed using BLASTn. The BLASTn results were parsed using a PERL script and imputed into the ACT program to construct synteny diagrams of the *MAT* locus alleles among *C. gattii* strains. In order to evaluate size polymorphisms within the *MAT* locus, 14 *MAT*α strains representing all *SXI1*α alleles and 8 *MAT* **a** strains were amplified using primers complementary to conserved sequences. Primers used for fingerprinting are listed in Table S3. The PCR products were digested using appropriate enzymes selected on the basis of DNA sequences using NEBcutter version 2.0 (http://tools.neb.com/NEBcutter2/index.php). Fingerprints showing variability were sequenced to determine the nature of any size polymorphisms. Sequences from both forward and reverse strands were assembled with complete double strand coverage and manually edited using Sequencher version 4.8 (Gene Codes Corporation).

### Murine virulence assays

The pathogenic potential of *C. gattii* strains belonging to the VGIII genotype was tested in a murine model of pulmonary cryptococcosis [97]. Briefly, *C. gattii* strains were grown in YPD broth for 16 hours and washed with sterile physiological saline. Cells were counted with a hemocytometer and suspended at a concentration of 3.3×10^6^ cells/ml. Five male BALB/c mice (approximately six-weeks old, 15–20 g; Charles River) in each group were first anesthetized with xylazine-ketamine mixture and then 30 ul of infectious inocula was gently dripped into their nares. The injected animals were observed for any overt signs of illness, and all morbid animals were promptly sacrificed by CO_2_ inhalation to minimize pain and suffering.

Data from all infected animals were used to determine Kaplan-Meyer survival curves using SAS software (SAS Institute, Inc., Cary, N.C.). For this statistical analysis with genotype information, the non-parametric Mann-Whitney U-test was applied, with further details in the statistical analysis section (http://faculty.vassar.edu/lowry/utest.html). Progression of disease was also determined by weighing infected animals once every alternate day at the start of the infection and then every day until they were moribund. One half portion of the lung and brain tissues from sacrificed animals was cultured on YPD and Staib's niger seed agar for the recovery of cells to determine that infections were cryptococcal in origin.

### Organ load and histopathology

To characterize virulence properties, representative *C. gattii* strains from four virulence groups comprising high virulence strains (CA1499), moderate virulence strains (CA1292), low virulence strains (WM161), and avirulent strains (CA2339), were chosen for further intranasal infection per procedure described previously. A total of six mice were infected for each test strain and groups of three mice were sacrificed at one and two-weeks post infection. The lungs and brains were removed aseptically, one half of each organ was fixed in 10% buffered formalin and Bouin's fixative, processed into paraffin blocks, sectioned, and stained with hematoxylin and eosin (H & E) and Mayer's mucicarmine for histopathological examination. The other halves of the lungs and brains were weighed, homogenized, diluted in PBS, and plated on YPD agar. Colonies were counted after incubation of the plates at 30°C for 4 days, and the results were expressed as colony forming unit (CFU) per gram of infected tissue. Results from organ load experiment were analyzed by student *t*-test, with significance determined at P≤0.05.

### Ethics statement

The animal studies conducted were in full compliance with all of the guidelines set forth by the Wadsworth Center Institutional Animal Care and Use Committee (IACUC) and in full compliance with the United States Animal Welfare Act (Public Law 98–198). The Wadsworth Center IACUC approved all of the vertebrate studies. The studies were conducted in facilities accredited by the Association for Assessment and Accreditation of Laboratory Animal Care (AAALAC).

### Intracellular Proliferation Rate (IPR) determination

A proliferation assay was previously developed to monitor the intracellular proliferation rate (IPR) of individual strains (for a 72-hour period) following phagocytosis [49]. For this assay, J774 macrophage cells were exposed to cryptococcal cells that were opsonized with 18B7 antibody for 2 hr as described previously [98]. Each well was washed with phosphate-buffered saline (PBS) in quadruplicate to remove as many extracellular yeast cells as possible and 1 ml of fresh serum-free DMEM was then added. For time point T = 0, the 1 ml of DMEM was discarded and 200 µl of sterile dH_2_O was added into wells to lyse macrophage cells. After 30 minutes, the intracellular yeast were released and collected. Another 200 µl dH_2_O was added to each well to collect the remaining yeast cells. The intracellular yeast were then mixed with Trypan Blue at a 1∶1 ratio and the live yeast cells were counted. For the subsequent five time points (T = 18 hrs, T = 24 hrs, T = 48 hrs, T = 72 hrs), intracellular cryptococcal cells were collected and independently counted with a hemocytometer.

For each strain tested, the time course was repeated at least three independent times, using different batches of macrophages. The IPR value was calculated by dividing the maximum intracellular yeast number by the initial intracellular yeast number at T = 0. We confirmed that Trypan Blue stains 100% of the cryptococcal cells in a heat-killed culture, but only approximately 5% of cells from a standard overnight culture. Compared to a conventional colony counting method, this method was shown to be more sensitive in detecting the clustered yeast population or yeast cells undergoing budding.

### Statistical analysis

Time to 50% lethality (LT50) and median IPR values were used to assess the statistical significance between the VGIII subgroups and these respective values. For this statistical analysis, the non-parametric directional Mann-Whitney U-test was applied and values of p<0.025 were considered as statistically significant (http://faculty.vassar.edu/lowry/utest.html). Regression analysis was used to measure the correlation between LT50 and IPR values, and an F-value of p<0.05 was considered to be a significant correlation, with R^2^, p values, and Pearson correlations also calculated and represented in the analysis.

### Strains

All strains used in this study are listed in Table S6, including *C. gattii*, *C. neoformans*, and *S. cerevisiae* isolates.

## Supporting Information

Figure S1 **A phylogenetic representation (NJ) and supporting bootstrap values of the sequence data from global VGIII isolates, with the exclusion of** ***MAT*** **locus linked markers (** ***SXI1*** **α/** ***SXI2*** **a).** VGI, VGII, and VGIV out-groups are included.
(TIFF)

Figure S2 **Additional haplotype networks for MLST markers not shown in** **Figure 4** **.** Alleles for each respective locus are indicated numerically. Orange coloration represents VGIIIa and green VGIIIb. Circles represent alleles extant in the population, and the smaller black circles represent alleles that have not been recovered, or which may no longer be extant in the population. Each line connected to an object represents one postulated evolutionary event, with the squared allele representing the posited ancestral allele.
(TIFF)

Figure S3 **Additional informative paired allele graphs from VGIII global isolates.** An hourglass shape indicates the presence of all four possible pairs of alleles and serves as evidence of recombination.
(PDF)

Figure S4 **Phenotypic analysis of VGIII** ***MAT*** **a isolates related to the loss of the** ***FCY1*** **gene from the** ***MAT*** **locus.** Results indicate that *FCY1* or another gene is still functioning to retain expected WT phenotypes involving sensitivity to the antifungal agent 5-Fluorocytosine (5-FC) and utilization of cytosine as a sole nitrogen source. Control strains include *S. cerevisiae* WT (S90) and an *fcy1*Δ mutant strain (S109) that is resistant to 5-FC and unable to utilize cytosine as a sole nitrogen source, and wild type control strains for *C. neoformans* var. *grubii* (H99) and *C. neoformans* var. *neoformans* (JEC21).
(TIFF)

Figure S5 **Molecular analysis of VGIII** ***MAT*** **a isolates related to the loss of the** ***FCY1*** **and** ***FAO1*** **genes from the** ***MAT*** **locus and the truncation of the** ***UAP1*** **gene.** A) List of strains. B) Southern blot analysis results indicate that the *FCY1* gene may no longer be present in the genome, or that the gene may have undergone accelerated evolution and thus not be detected by hybridization to the probe that is based on the VGIIIα (NIH312) gene sequence. C) *GPD1* control for Southern blot. D–F) PCR analysis for *FCY1*, *FAO1*, and *UAP1*, respectively, indicating a loss of the genes from the VGIII *MAT* **a** genomes, or that the genes might have undergone accelerated evolution and thus not be detected with the oligonucleotide primers used.
(TIFF)

Figure S6 **Remnant of** ***SXI2*** **a in VGIII α isolates. A) The grey shading indicates intergenic** ***MAT*** **sequence, while the black line indicates sequence outside of the** ***MAT*** **locus allele.** The homology of the *SXI2* **a** remnant is indicated on the figure. B) A model for the evolution of the remnant based on partial gene loss in *C. gattii* and complete gene loss in *C. neoformans*. C) A model for the evolution of the remnant based on gene conversion within *C. gattii*.
(TIFF)

Table S1 **Summary of mating assays and fertility.** Top four isolates for fertility from each mating type indicate isolates that mated with the largest number of opposite mating type partners.
(XLSX)

Table S2 **Details of fingerprinting regions with digestion information for** ***MAT*** **PCR products that were subject to restriction enzyme digestion.**
(XLSX)

Table S3 **Primers used in the study.**
(XLSX)

Table S4 **GenBank accession numbers for all of the MLST alleles represented in the text and figures, and sequences of the mating type loci.**
(XLSX)

Table S5 **Detailed sequence type information from** **Figure 3** **and Figure S1.**
(XLSX)

Table S6 **Strains used in this study.**
(XLSX)
