# Correction: A Diverse Population of *Cryptococcus gattii* Molecular Type VGIII in Southern Californian HIV/AIDS Patients

**DOI:** 10.1371/annotation/a23709b0-8d67-4b57-aa05-d5d70d830724

**Published:** 2011-11-03

**Authors:** Edmond J. Byrnes, Wenjun Li, Ping Ren, Yonathan Lewit, Kerstin Voelz, James A. Fraser, Fred S. Dietrich, Robin C. May, Sudha Chaturvedi, Vishnu Chaturvedi, Joseph Heitman

The ninth and tenth author's names were spelled incorrectly. The correct names are: Sudha Chaturvedi and Vishnu Chaturvedi. 

