# Supplementary figures and images for: A Diverse Population of Cryptococcus gattii Molecular Type VGIII in Southern Californian HIV/AIDS Patients
Source: PLoS Pathog. 2011 Sep 1;7(9):e1002205. doi: 10.1371/journal.ppat.1002205 (PMC3164645; doi:10.1371/journal.ppat.1002205)

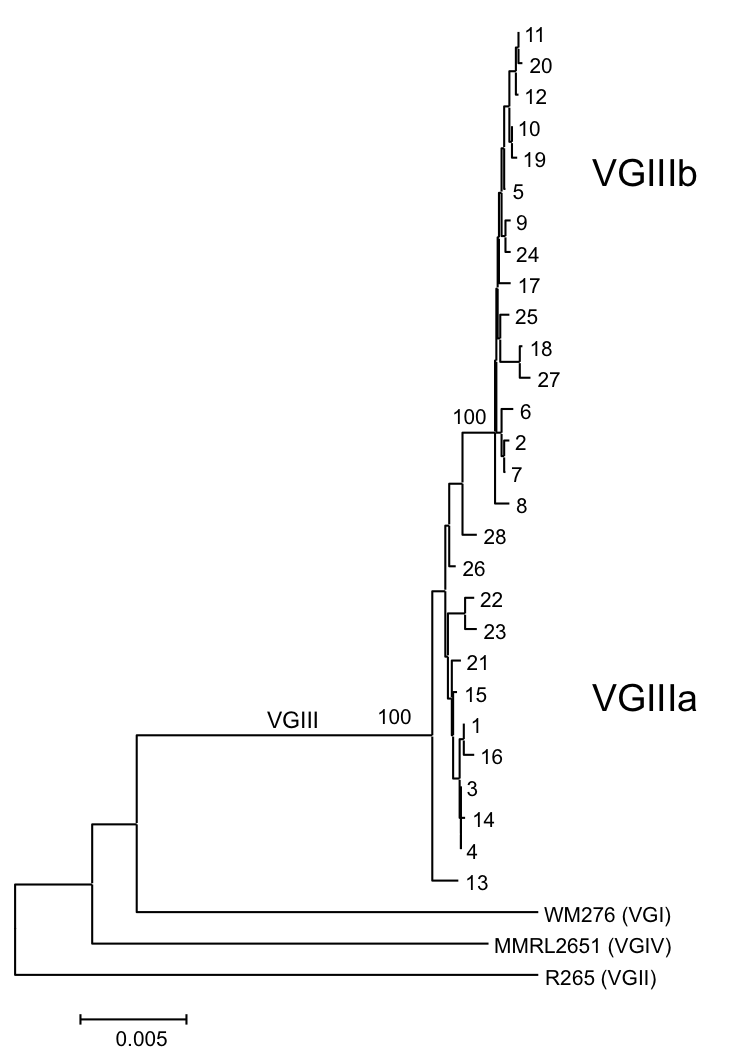

Supplement: Figure S1 — A phylogenetic representation (NJ) and supporting bootstrap values of the sequence data from global VGIII isolates, with the exclusion of MAT locus linked markers ( SXI1 α/ SXI2 a). VGI, VGII, and VGIV out-groups are included. (TIFF) [file ppat.1002205.s001.tiff]

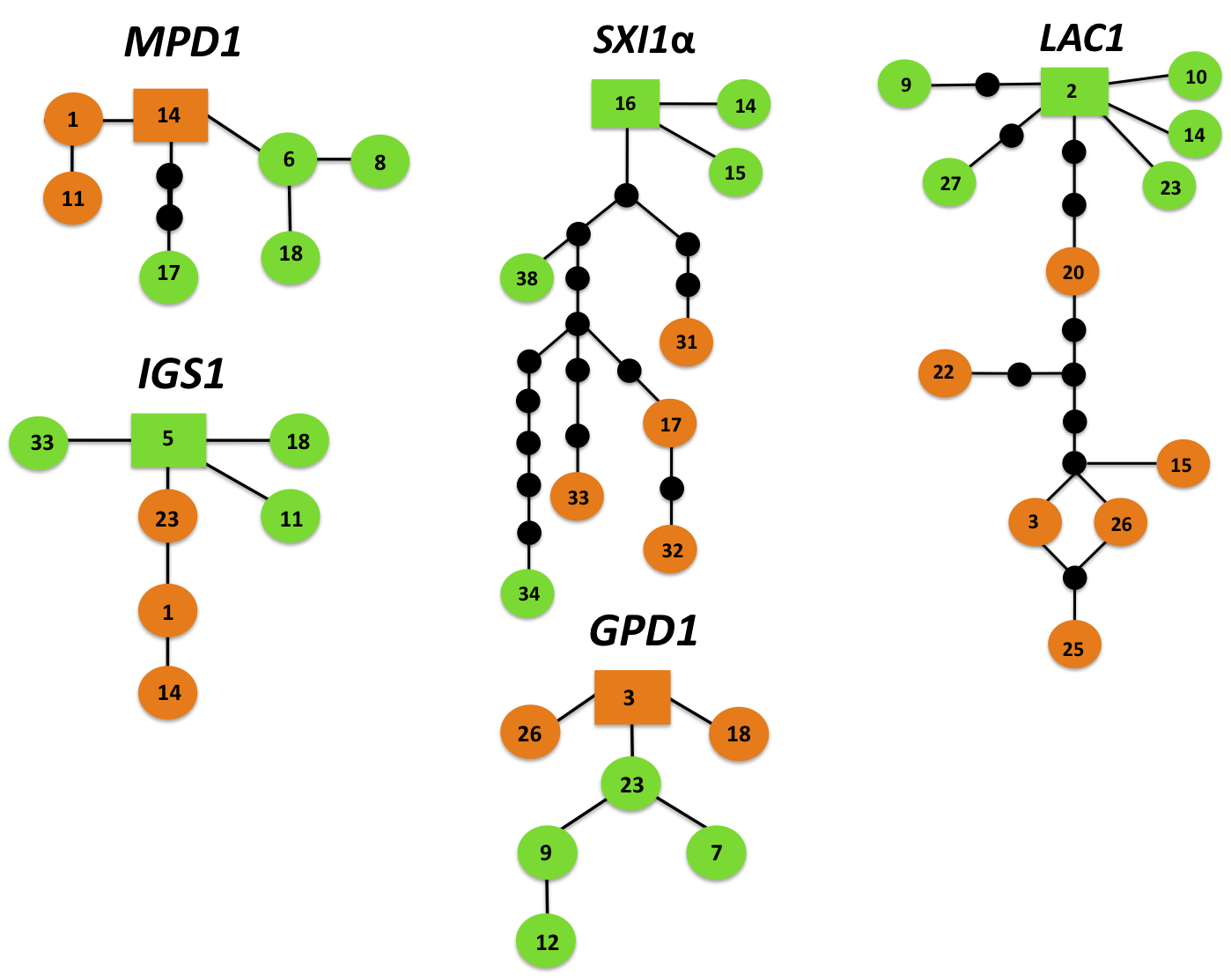

Supplement: Figure S2 — Additional haplotype networks for MLST markers not shown in Figure 4 . Alleles for each respective locus are indicated numerically. Orange coloration represents VGIIIa and green VGIIIb. Circles represent alleles extant in the population, and the smaller black circles represent alleles that have not been recovered, or which may no longer be extant in the population. Each line connected to an object represents one postulated evolutionary event, with the squared allele representing the posited ancestral allele. (TIFF) [file ppat.1002205.s002.tiff]

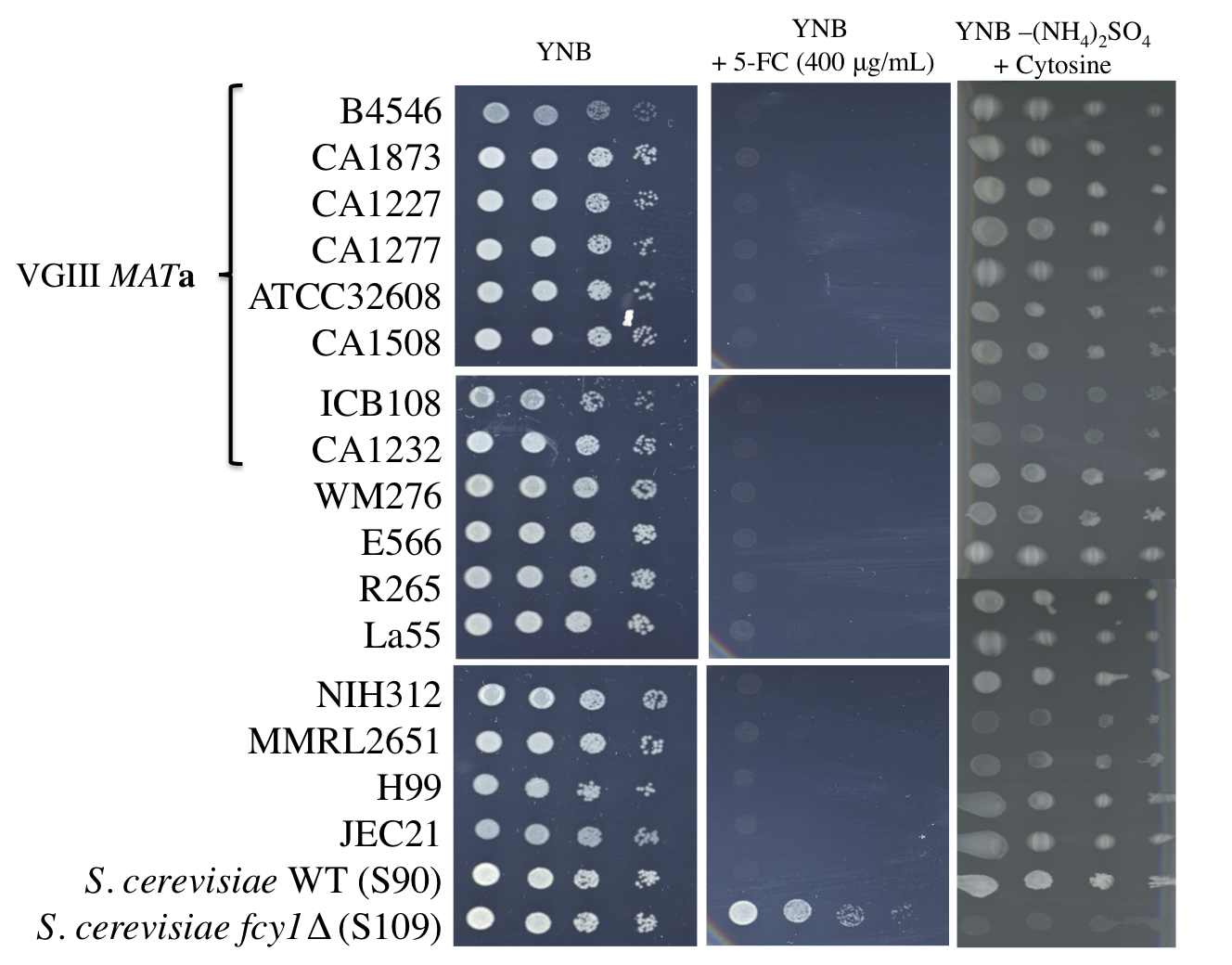

Supplement: Figure S4 — Phenotypic analysis of VGIII MAT a isolates related to the loss of the FCY1 gene from the MAT locus. Results indicate that FCY1 or another gene is still functioning to retain expected WT phenotypes involving sensitivity to the antifungal agent 5-Fluorocytosine (5-FC) and utilization of cytosine as a sole nitrogen source. Control strains include S. cerevisiae WT (S90) and an fcy1Δ mutant strain (S109) that is resistant to 5-FC and unable to utilize cytosine as a sole nitrogen source, and wild type control strains for C. neoformans var. grubii (H99) and C. neoformans var. neoformans (JEC21). (TIFF) [file ppat.1002205.s004.tiff]

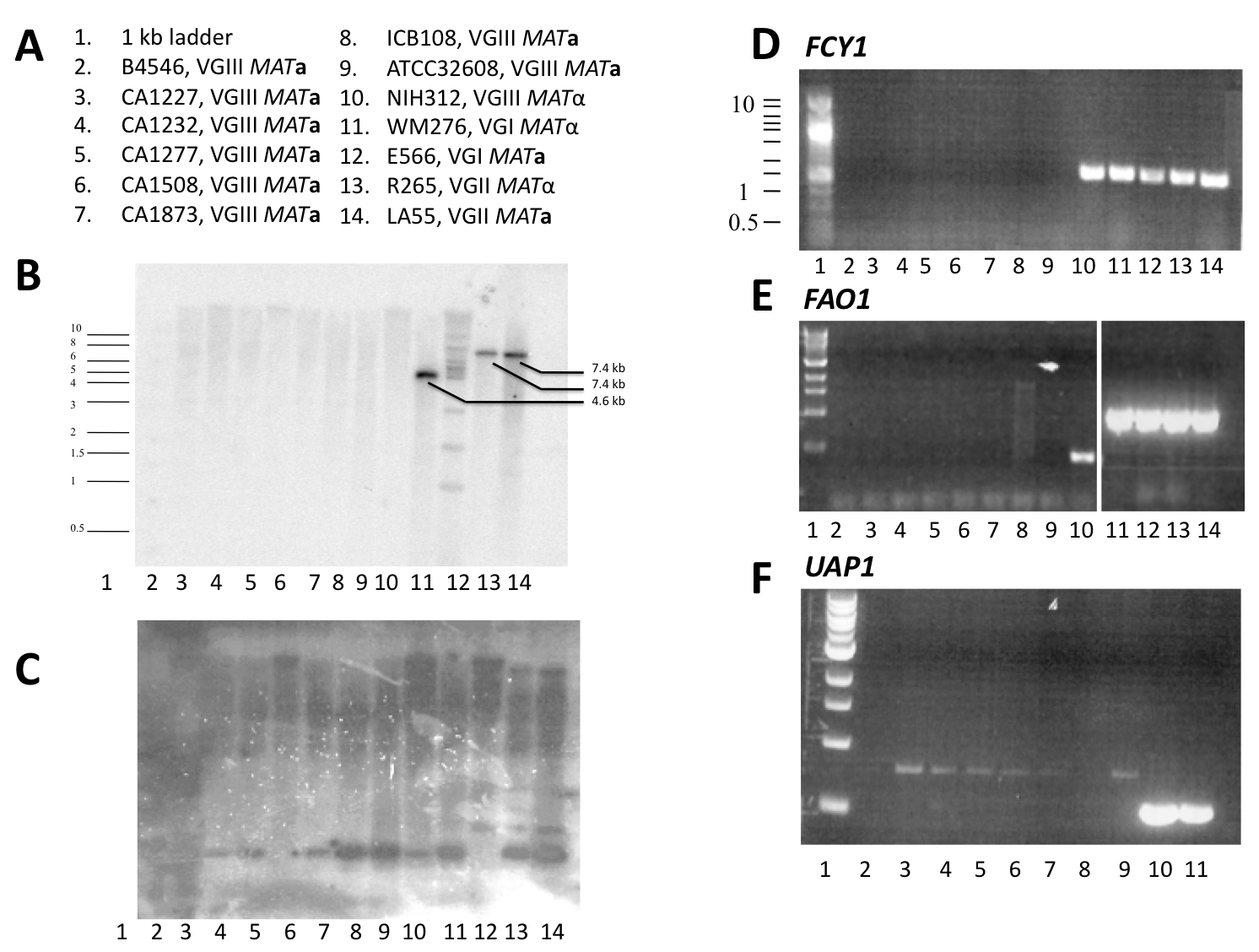

Supplement: Figure S5 — Molecular analysis of VGIII MAT a isolates related to the loss of the FCY1 and FAO1 genes from the MAT locus and the truncation of the UAP1 gene. A) List of strains. B) Southern blot analysis results indicate that the FCY1 gene may no longer be present in the genome, or that the gene may have undergone accelerated evolution and thus not be detected by hybridization to the probe that is based on the VGIIIα (NIH312) gene sequence. C) GPD1 control for Southern blot. D–F) PCR analysis for FCY1, FAO1, and UAP1, respectively, indicating a loss of the genes from the VGIII MAT a genomes, or that the genes might have undergone accelerated evolution and thus not be detected with the oligonucleotide primers used. (TIFF) [file ppat.1002205.s005.tiff]

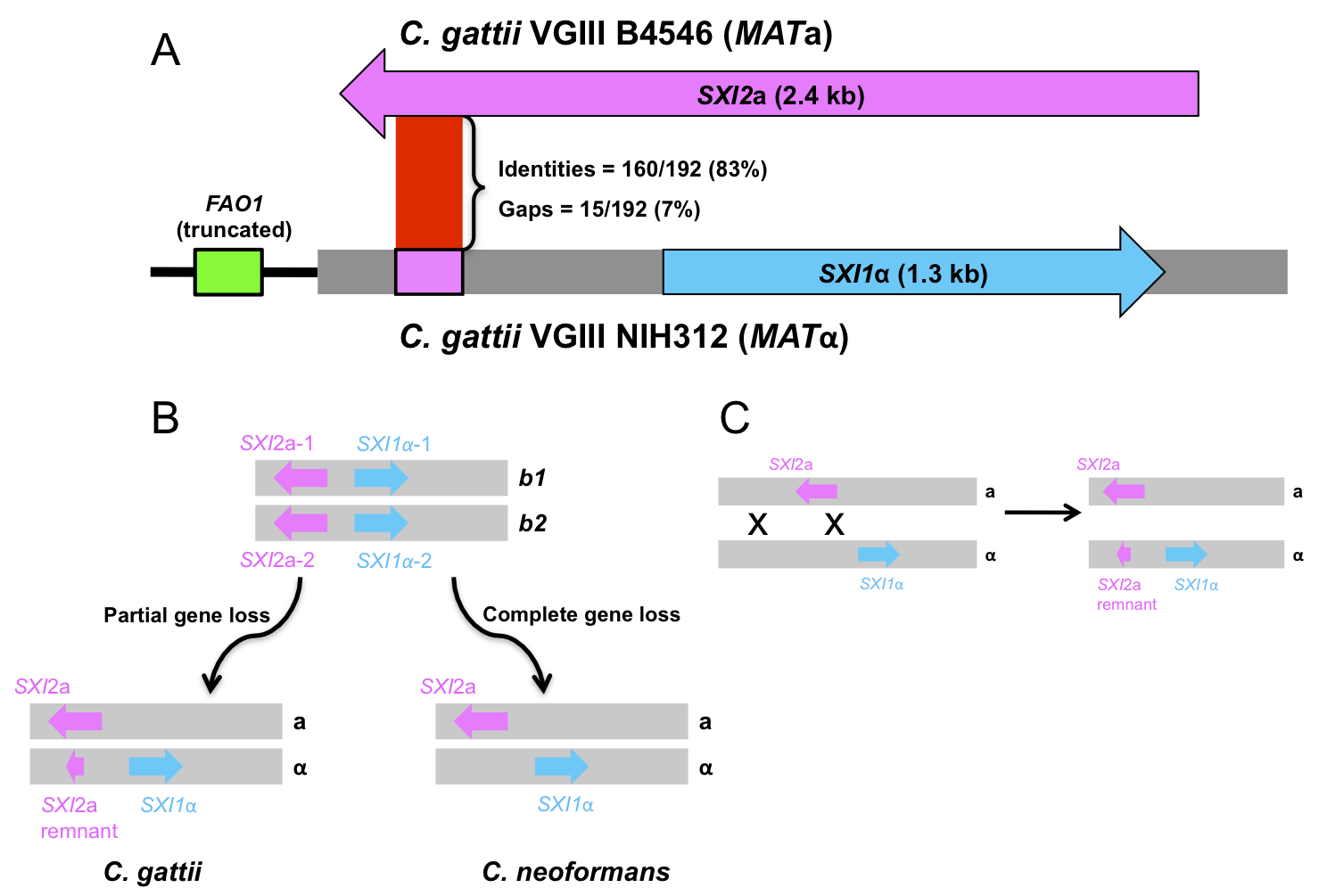

Supplement: Figure S6 — Remnant of SXI2 a in VGIII α isolates. A) The grey shading indicates intergenic MAT sequence, while the black line indicates sequence outside of the MAT locus allele. The homology of the SXI2 a remnant is indicated on the figure. B) A model for the evolution of the remnant based on partial gene loss in C. gattii and complete gene loss in C. neoformans. C) A model for the evolution of the remnant based on gene conversion within C. gattii. (TIFF) [file ppat.1002205.s006.tiff]
