# Supplementary material for: A Diverse Population of Cryptococcus gattii Molecular Type VGIII in Southern Californian HIV/AIDS Patients
Source: PLoS Pathog. 2011 Sep 1;7(9):e1002205. doi: 10.1371/journal.ppat.1002205 (PMC3164645; doi:10.1371/journal.ppat.1002205)

*SXI1*α vs. *IGS*

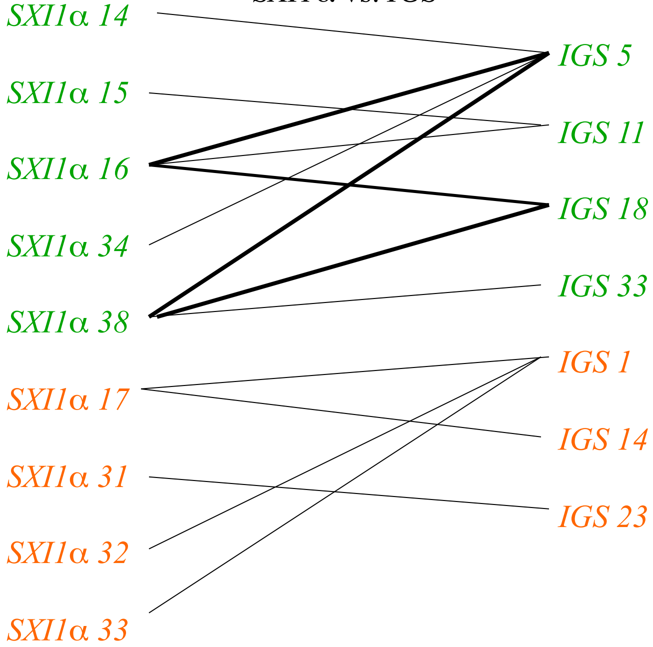

*SXI1*α vs. *TEF1*

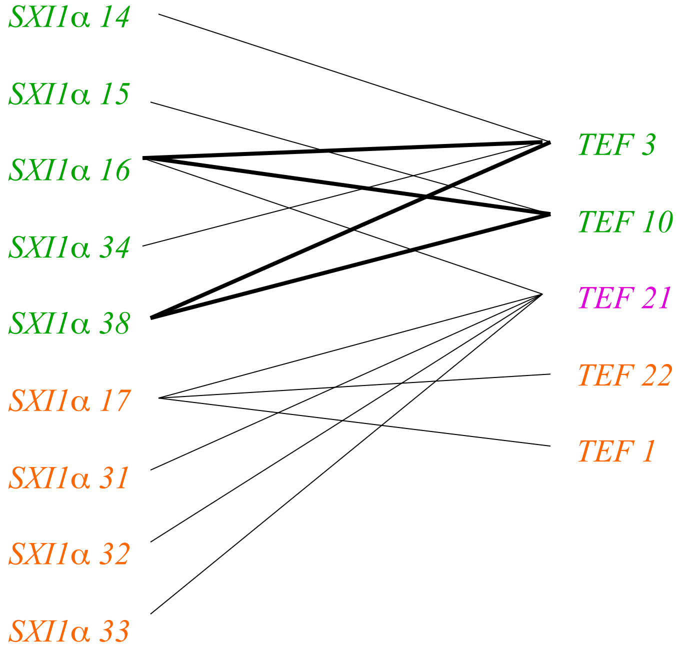

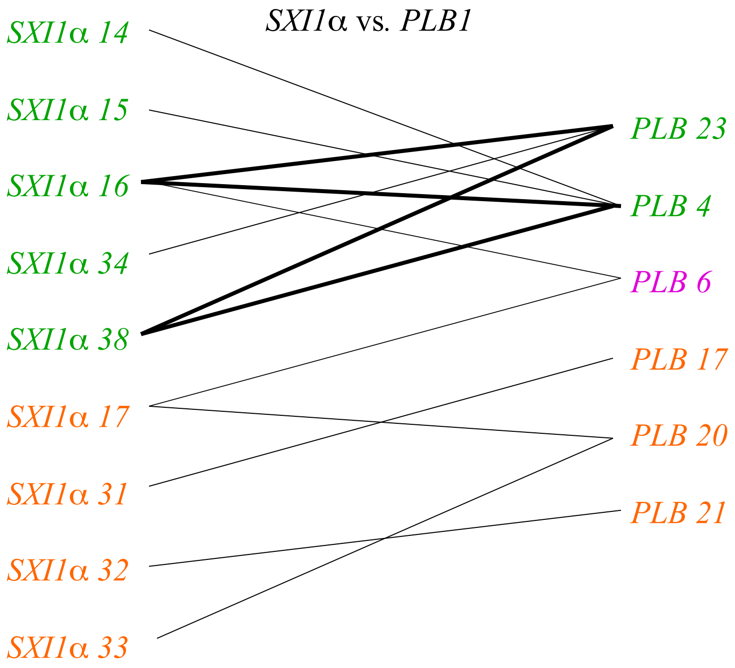
SXI1α vs. PLB1
SXI1α 14
SXI1α 15
SXI1α 16
SXI1α 34
SXI1α 38
SXI1α 17
SXI1α 31
SXI1α 32
SXI1α 33
PLB 23
PLB 4
PLB 6
PLB 17
PLB 20
PLB 21

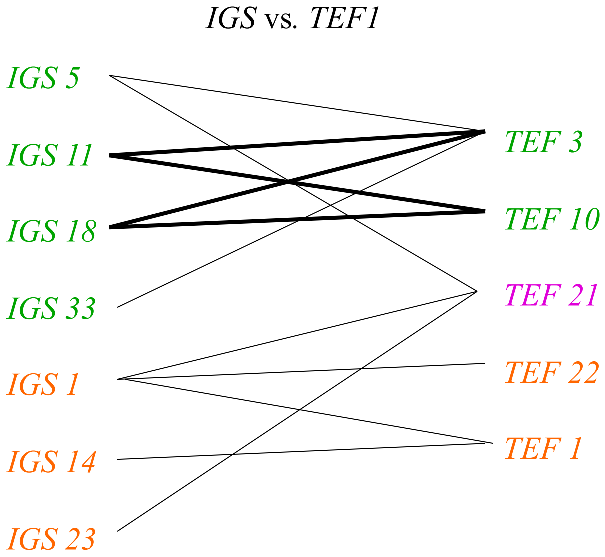
IGS vs. TEF1
IGS 5
IGS 11
IGS 18
IGS 33
IGS 1
IGS 14
IGS 23
TEF 3
TEF 10
TEF 21
TEF 22
TEF 1

IGS vs. *GPD1*

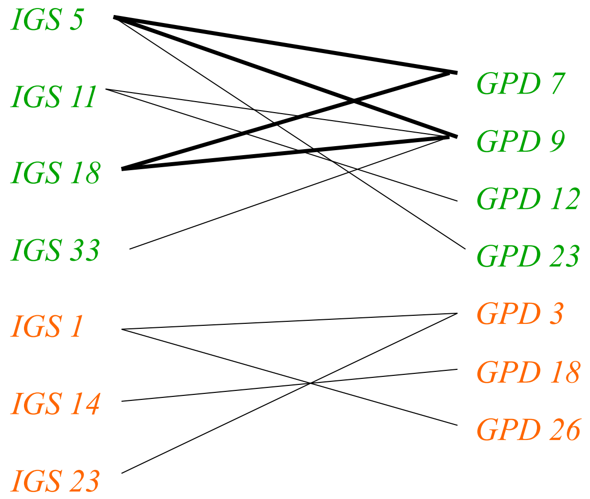

IGS vs. *LAC1*

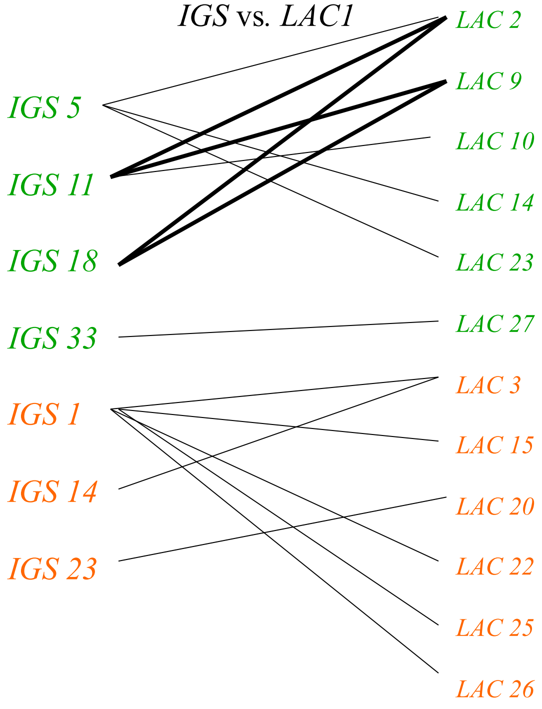

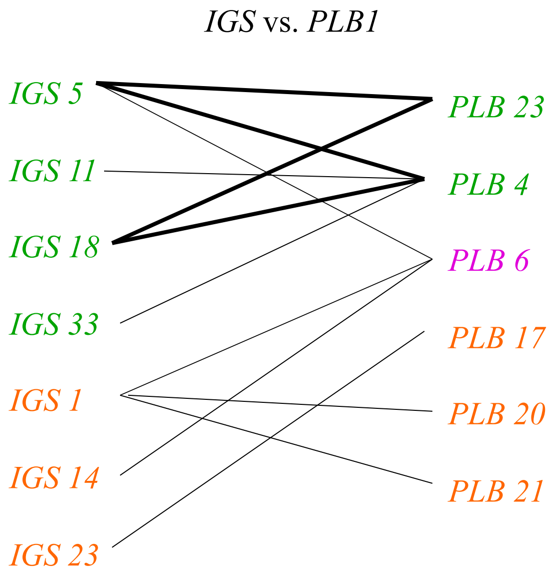
IGS vs. PLB1
IGS 5
IGS 11
IGS 18
IGS 33
IGS 1
IGS 14
IGS 23
PLB 23
PLB 4
PLB 6
PLB 17
PLB 20
PLB 21

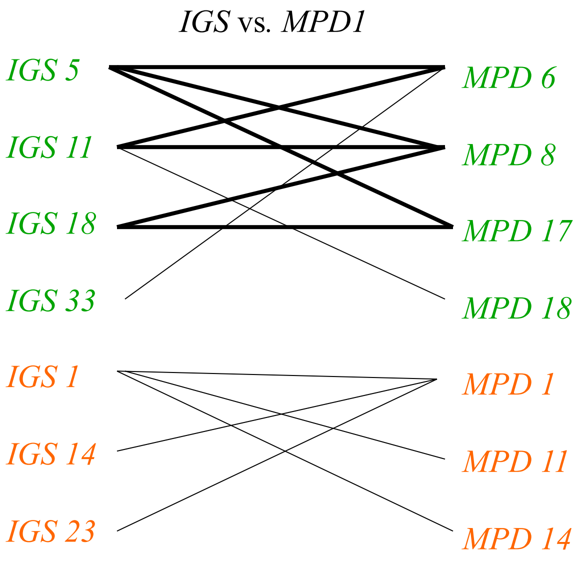
IGS vs. MPD1
IGS 5
IGS 11
IGS 18
IGS 33
IGS 1
IGS 14
IGS 23
MPD 6
MPD 8
MPD 17
MPD 18
MPD 1
MPD 11
MPD 14

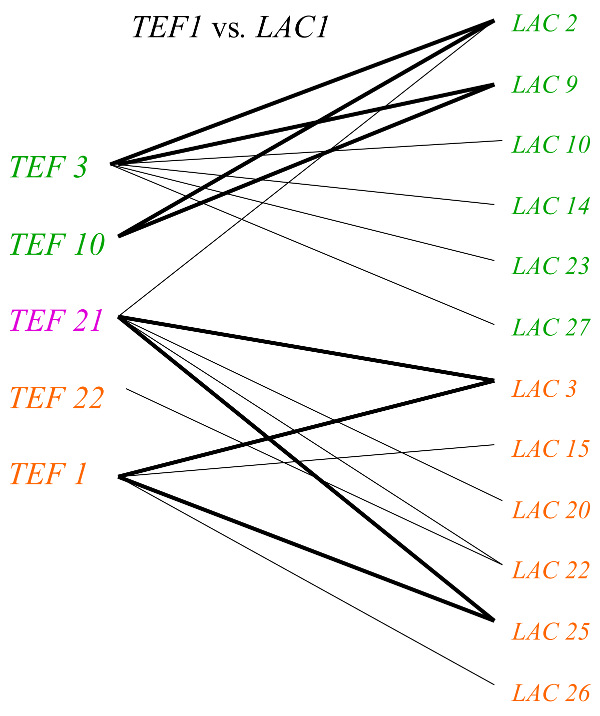
TEF1 vs. LAC1
TEF 3
TEF 10
TEF 21
TEF 22
TEF 1
LAC 2
LAC 9
LAC 10
LAC 14
LAC 23
LAC 27
LAC 3
LAC 15
LAC 20
LAC 22
LAC 25
LAC 26

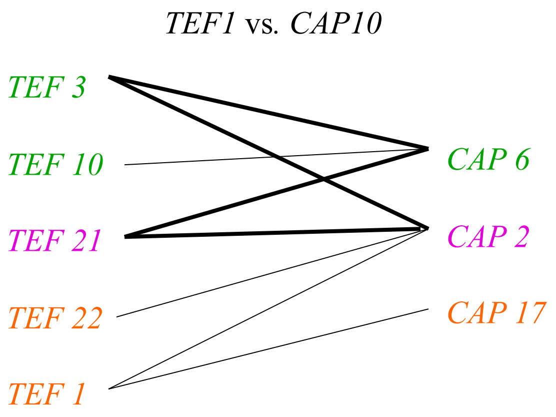
TEF1 vs. CAP10
TEF 3
TEF 10
TEF 21
TEF 22
TEF 1
CAP 6
CAP 2
CAP 17

TEF1 vs. *PLB1*

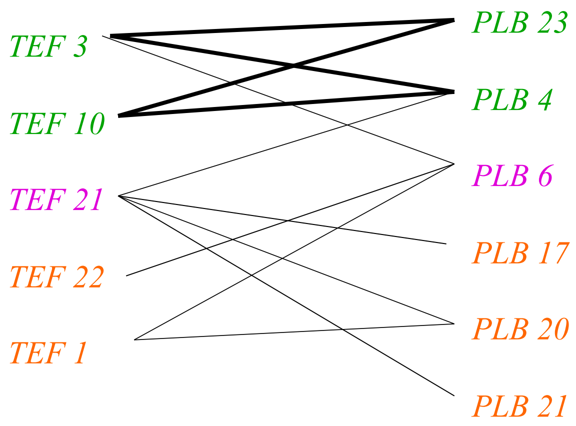

TEF1 vs. *MPD1*

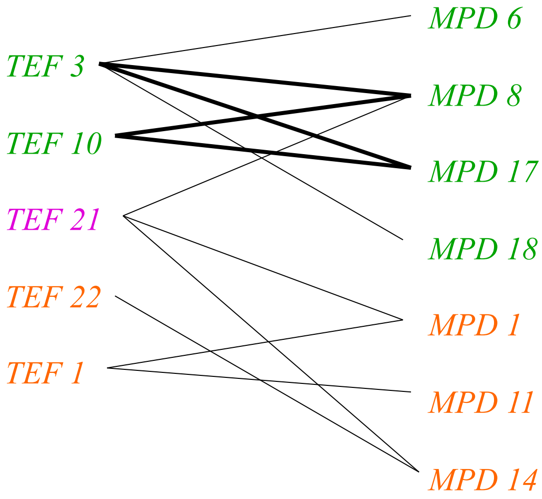

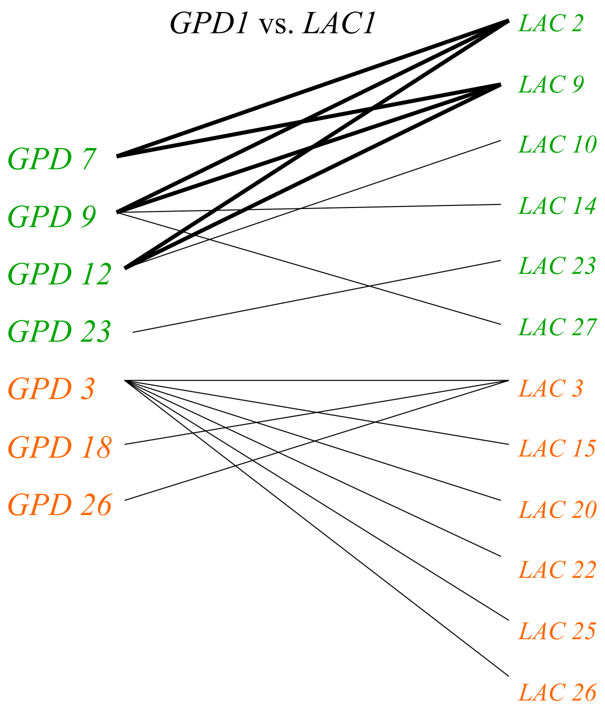
GPD1 vs. LAC1
GPD 7
GPD 9
GPD 12
GPD 23
GPD 3
GPD 18
GPD 26
LAC 2
LAC 9
LAC 10
LAC 14
LAC 23
LAC 27
LAC 3
LAC 15
LAC 20
LAC 22
LAC 25
LAC 26

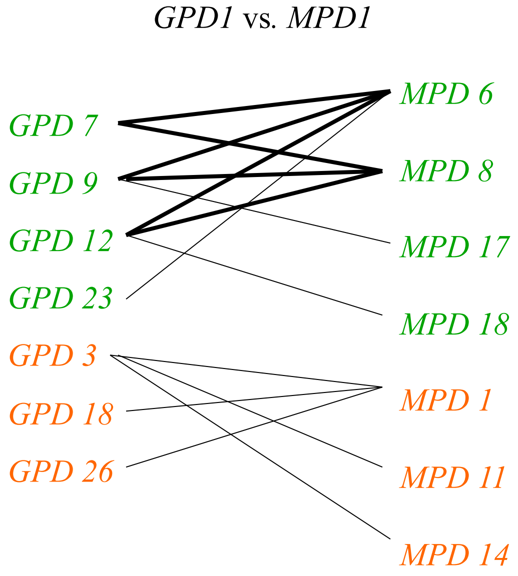
GPD1 vs. MPD1
GPD 7
GPD 9
GPD 12
GPD 23
GPD 3
GPD 18
GPD 26
MPD 6
MPD 8
MPD 17
MPD 18
MPD 1
MPD 11
MPD 14

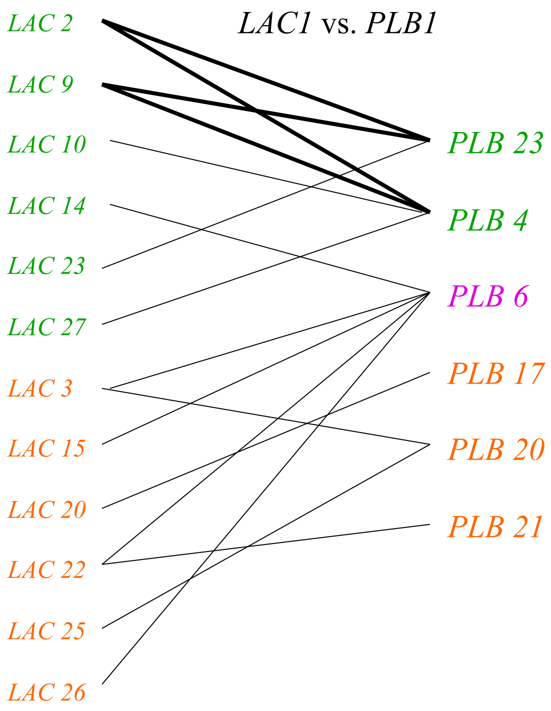
LAC1 vs. PLB1
LAC 2
LAC 9
LAC 10
LAC 14
LAC 23
LAC 27
LAC 3
LAC 15
LAC 20
LAC 22
LAC 25
LAC 26
PLB 23
PLB 4
PLB 6
PLB 17
PLB 20
PLB 21

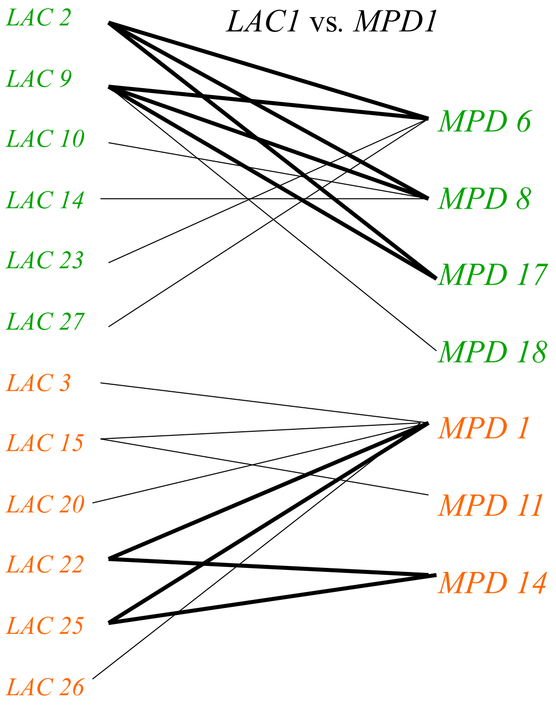
LAC1 vs. MPD1
LAC 2
LAC 9
LAC 10
LAC 14
LAC 23
LAC 27
LAC 3
LAC 15
LAC 20
LAC 22
LAC 25
LAC 26
MPD 6
MPD 8
MPD 17
MPD 18
MPD 1
MPD 11
MPD 14

CAP10 vs. *PLB1*

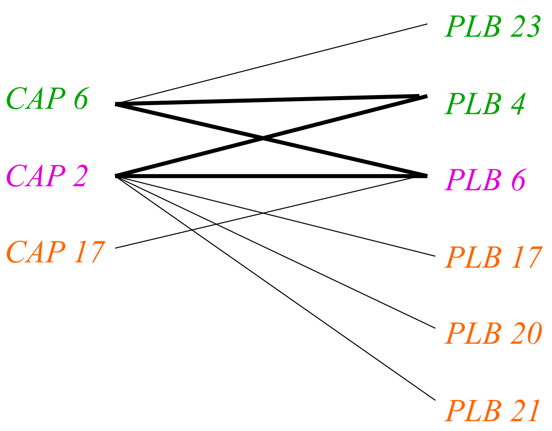

PLB1 vs. *MPD1*

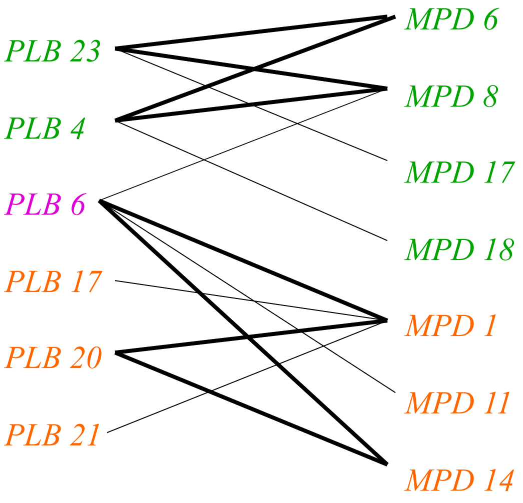

Supplement: Figure S3 — Additional informative paired allele graphs from VGIII global isolates. An hourglass shape indicates the presence of all four possible pairs of alleles and serves as evidence of recombination. (PDF) [file ppat.1002205.s003.pdf]
